# Supplementary material for: Dynamics of GLP-1R peptide agonist engagement are correlated with kinetics of G protein activation
Source: Nat Commun. 2022 Jan 10;13:92. doi: 10.1038/s41467-021-27760-0 (PMC8748714; doi:10.1038/s41467-021-27760-0)
Supplement: Supplementary file 1 — Supplementary Information [file 41467_2021_27760_MOESM1_ESM.pdf]

Supplementary Information

Dynamics of GLP-1R peptide agonist engagement are correlated with kinetics of G protein activation

Giuseppe Deganutti[1,2*], Yi-Lynn Liang[3^*], Xin Zhang[3,4*], Maryam Khoshouei[5&*], Lachlan Clydesdale[3*], Matthew J. Belousoff[3,4], Hari Venugopal[6], Tin T. Truong[3], Alisa Glukhova[3$], Andrew N. Keller[3], Karen J. Gregory[3], Katie Leach[3], Arthur Christopoulos[3,4], Radostin Danev[7], Christopher A. Reynolds[1,2¶], Peishen Zhao[3,4¶], Patrick M. Sexton[3,4¶], Denise Wootten[3,4¶]

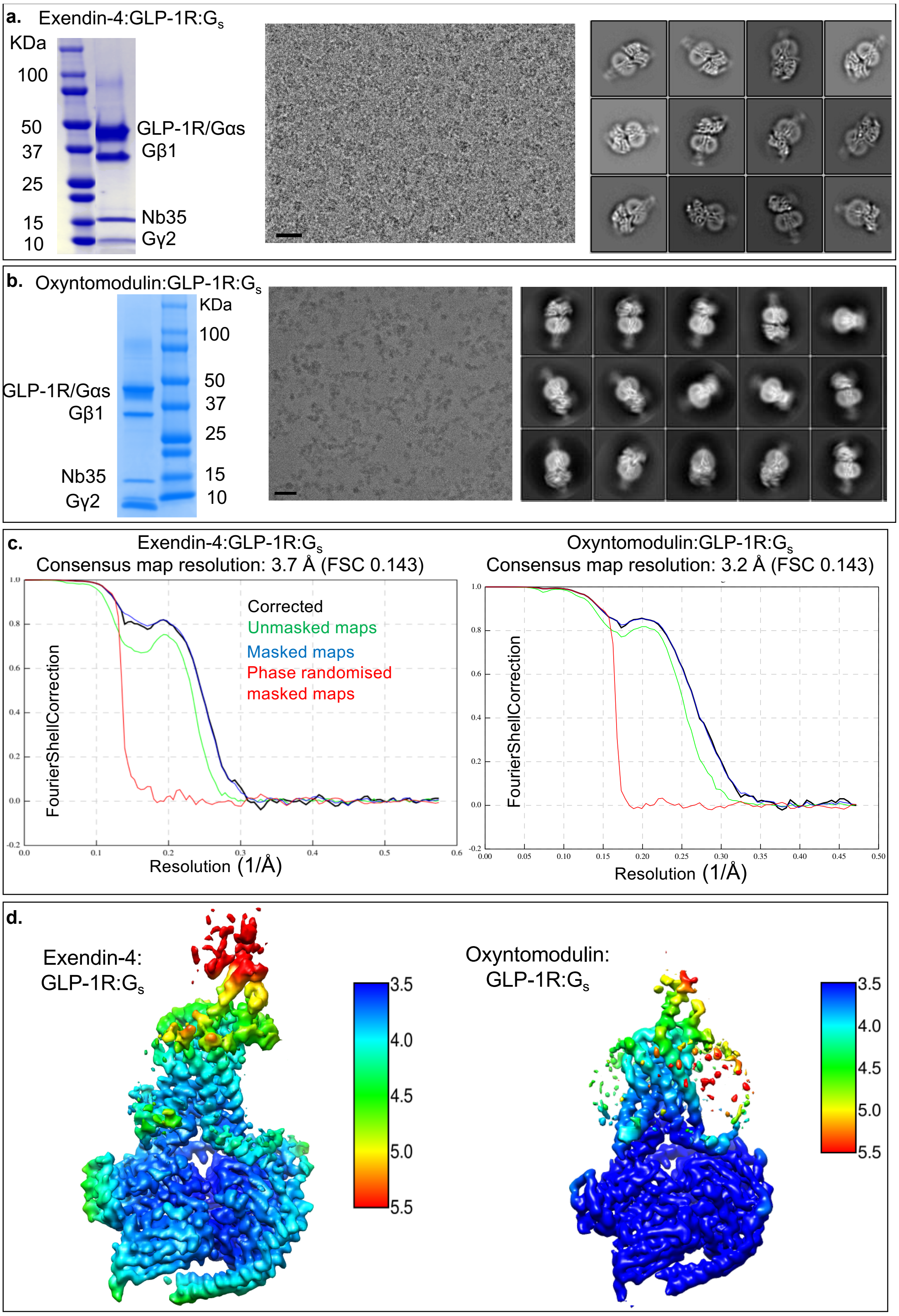

Supplementary Figure 1. Purification, cryo-EM data imaging and processing of exendin-4 and oxyntomodulin bound GLP-1R:Gs complexes. a, Exendin-4 bound complex. Left; Coomassie stained gel showing the prep purity containing all expected components. Middle; Representative micrograph (of 8816) of the exendin-4:GLP-1R:Gs complex (scale bar 20 nM). Right; Two-dimensional classaverages of the complex in LMNG micelle. b, oxyntomodulin bound complex. Left; Coomassie stained gel showing the prep purity containing all expected components. Middle; Representative micrograph (of 2364) of the oxyntomodulin:GLP-1R:Gs complex (scale bar 20 nM). Right; Two-dimensional class averages of the complex in LMNG micelle. c, "Gold standard" Fourier shell correlation (FSC) curves for the exendin-4 bound complex (left) and the oxyntomodulin bound complex (right), showing the overall nominal resolution at 3.7 Å and 3.3 Å, respectively. d, Cryo-EM density maps for the exendin-4 complex (left) and the oxyntomodulin bound complex (right), coloured according to local resolution (scaled in Å; dark blue, highest resolution, red, lowest resolution).

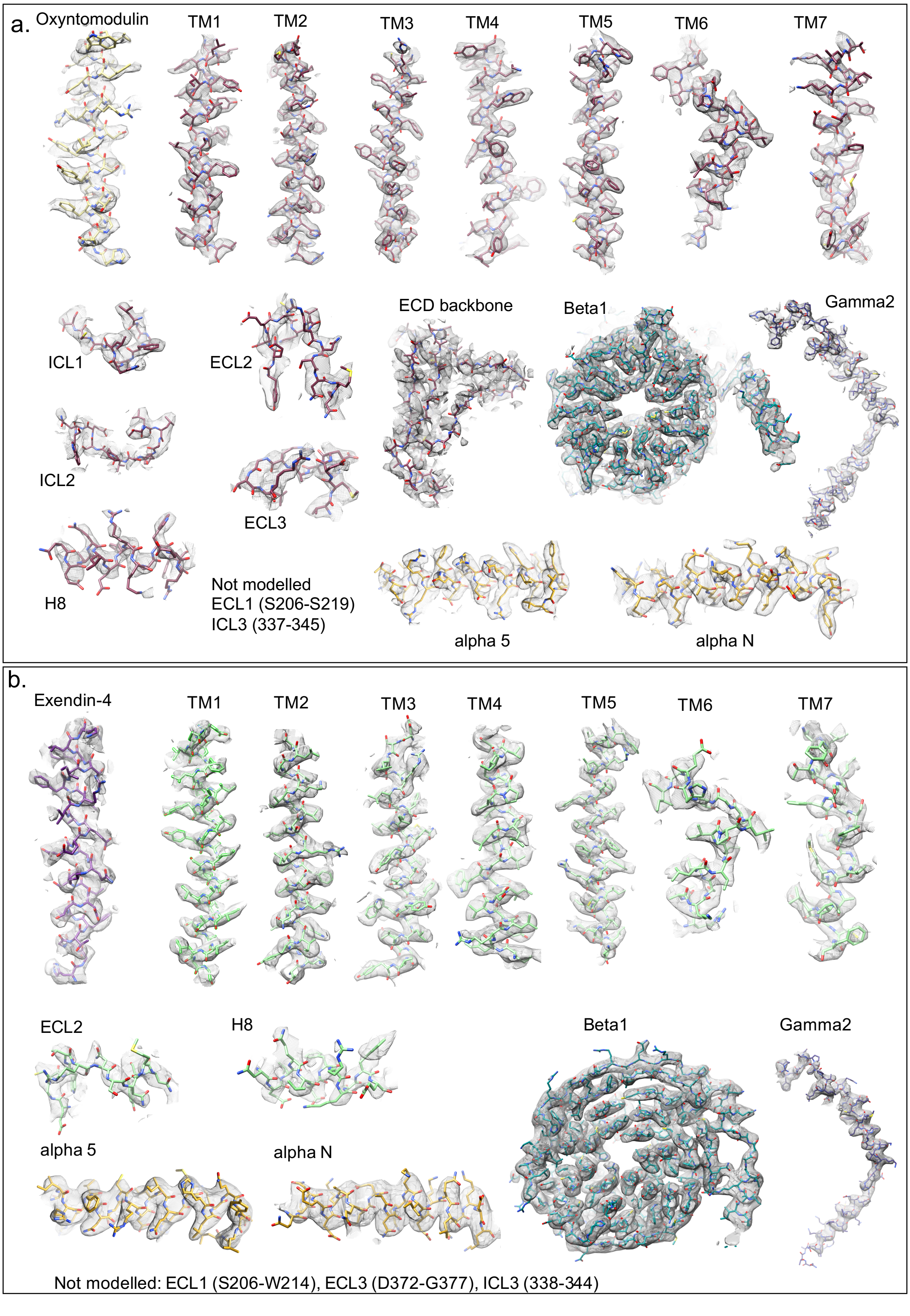

Supplementary Figure 2. The atomic resolution model of the exendin-4-bound and oxyntomodulin bound GLP-1R:Gs in the cryo-EM density map. EM density map and the model are shown for all seven TM helices and H8 of the receptor, ECLs and ICLs where modelled, the α5 and α5 helices of the GαS Ras-like domain, b1 and g2 and the peptide agonists for the oxyntomodulin (a) and exendin-4 (b) bound complexes. The ECD density to model fit is also shown for the oxyntomodulin sample.

Supplementary Figure 3.

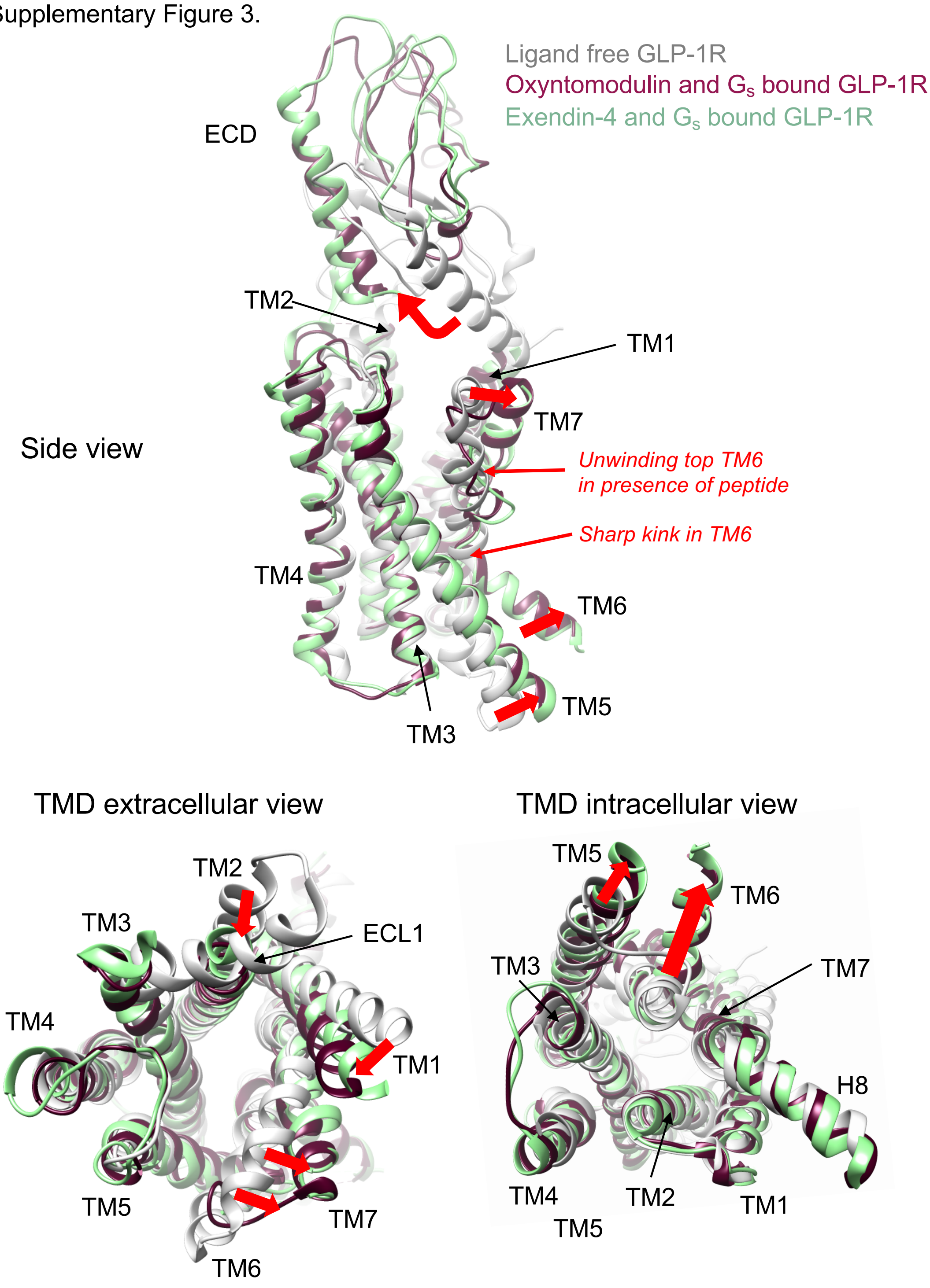

Supplementary Figure 3. GLP-1R conformational transitions from the inactive conformation to Gs bound conformations induced by exendin-4 and oxyntomodulin. Superimposition of the inactive ligand free GLP-1R structure (6LN230 –pale grey) with the GLP-1R in its Gs bound conformation induced by oxyntomodulin (dark pink) and exendin-4 (pale green). Top; full length receptors viewed from the side. Bottom left; TMD viewed from the extracellular face. Bottom right; TMD viewed from the intracellular face. Red arrows/labels highlight the most substantial backbone conformational transitions between the inactive (grey) and the activated peptide bound (dark pink/pale green) GLP-1R conformations.

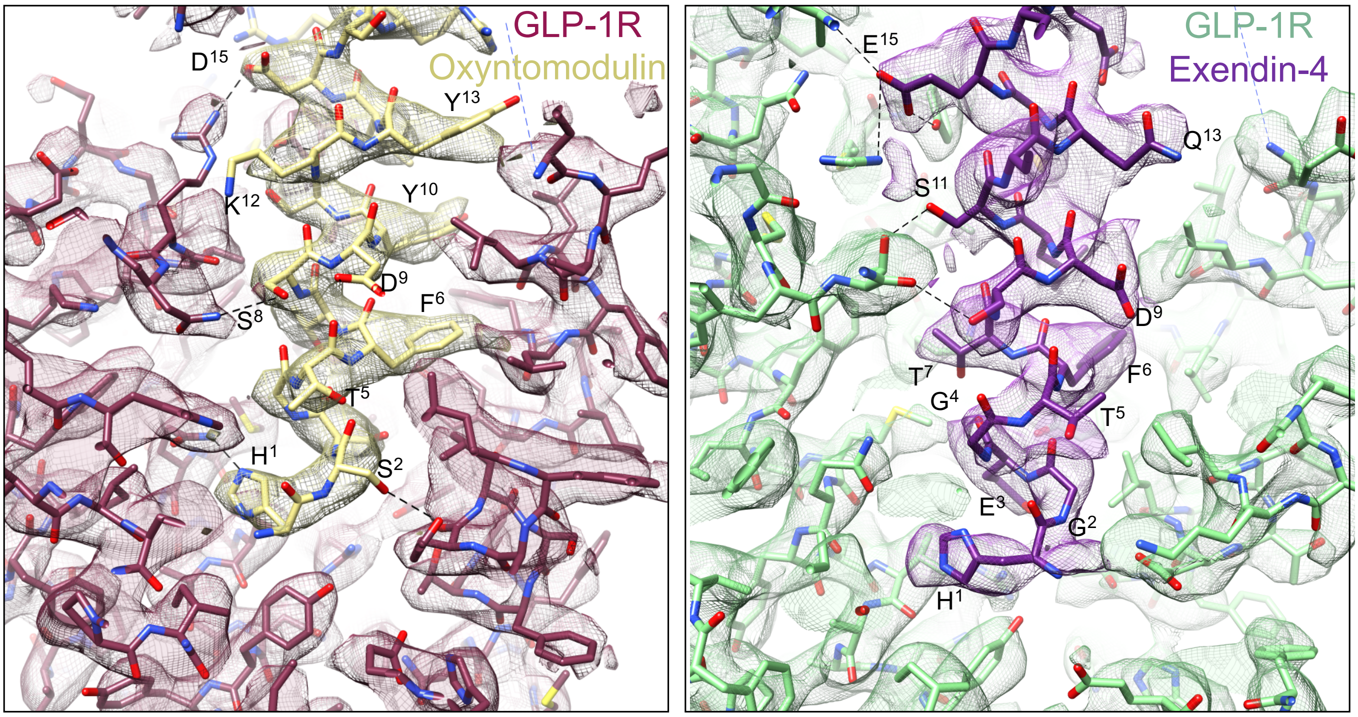

Supplementary Figure 4. Cut through of the peptide binding cavity in the TMD showing the models and cryo-EM maps. Cut-through of the TMD peptide binding cavity highlighting the density on the peptide and the interacting residues for the oxyntomodulin bound GLP-1R (left) and the exendin-4 bound GLP-1R (right). Residues in the peptide are labelled. Linked to Figure 2 where peptide and interacting TMD residues are labelled.

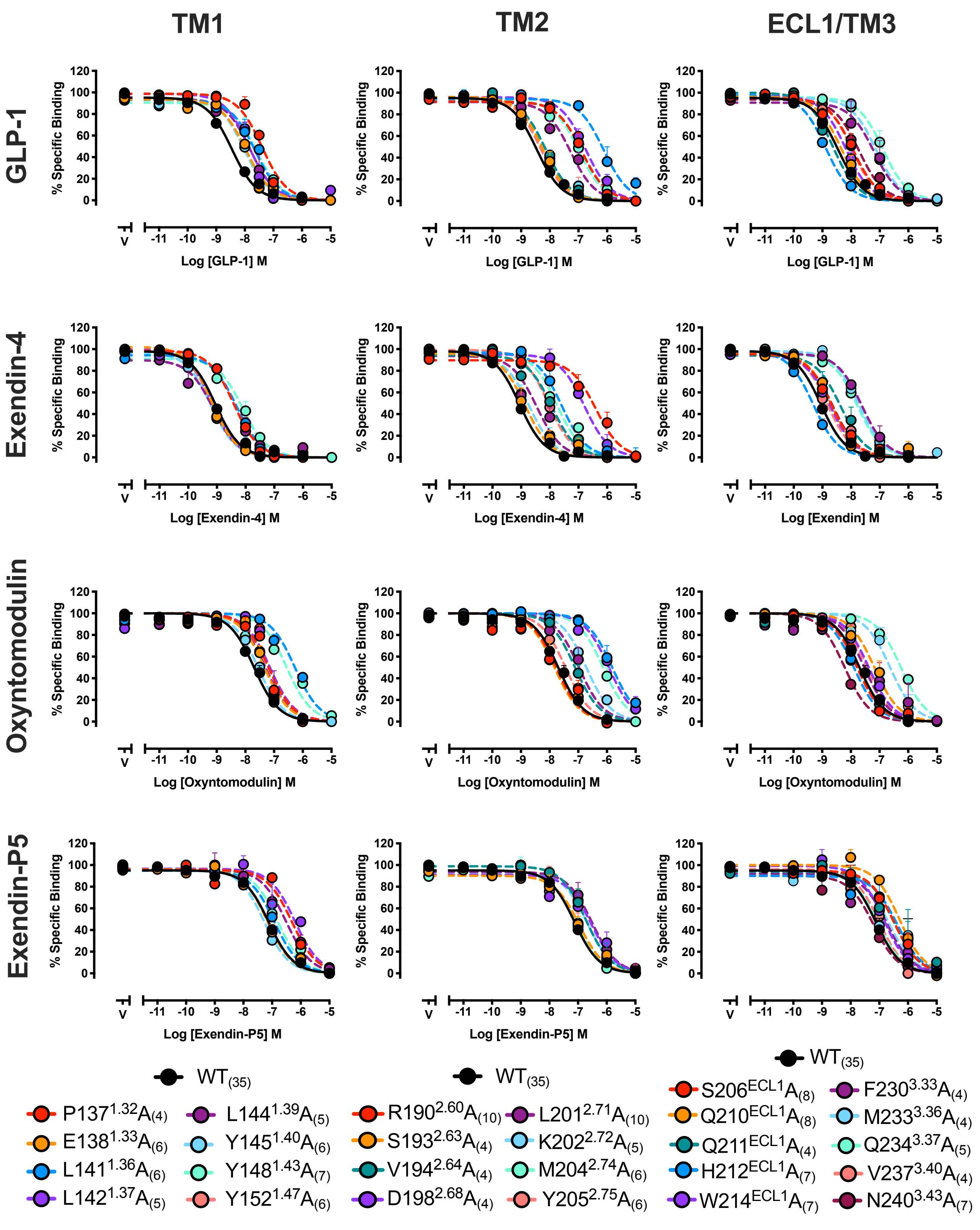

Supplementary Figure 5. Inhibition binding curves for wildtype and alanine mutants of GLP-1R residues within TMs 1-3. Equilibrium competition binding assays assessing the ability of GLP-1, oxyntomodulin, exendin-4 and exendin-P5 to displace the radioligand ^{125}I-exendin(9-39), in Cho-FlpIn cells overexpressing wildtype or mutant GLP-1Rs. Data are presented as % specific binding with 100% binding defined as total probe binding in the absence of competing ligand and non-specific (0%) binding determined as probe binding in the presence of 10 μM exendin(9-39). Data are means + s.e.m. of 4-10 independent experiments for mutant receptors performed in duplicate. For the GLP-1 dataset, the exact no of individual experiments are highlighted in parenthesis and subscript on the figure for each receptor construct. Source data are provided in the Source Data file.

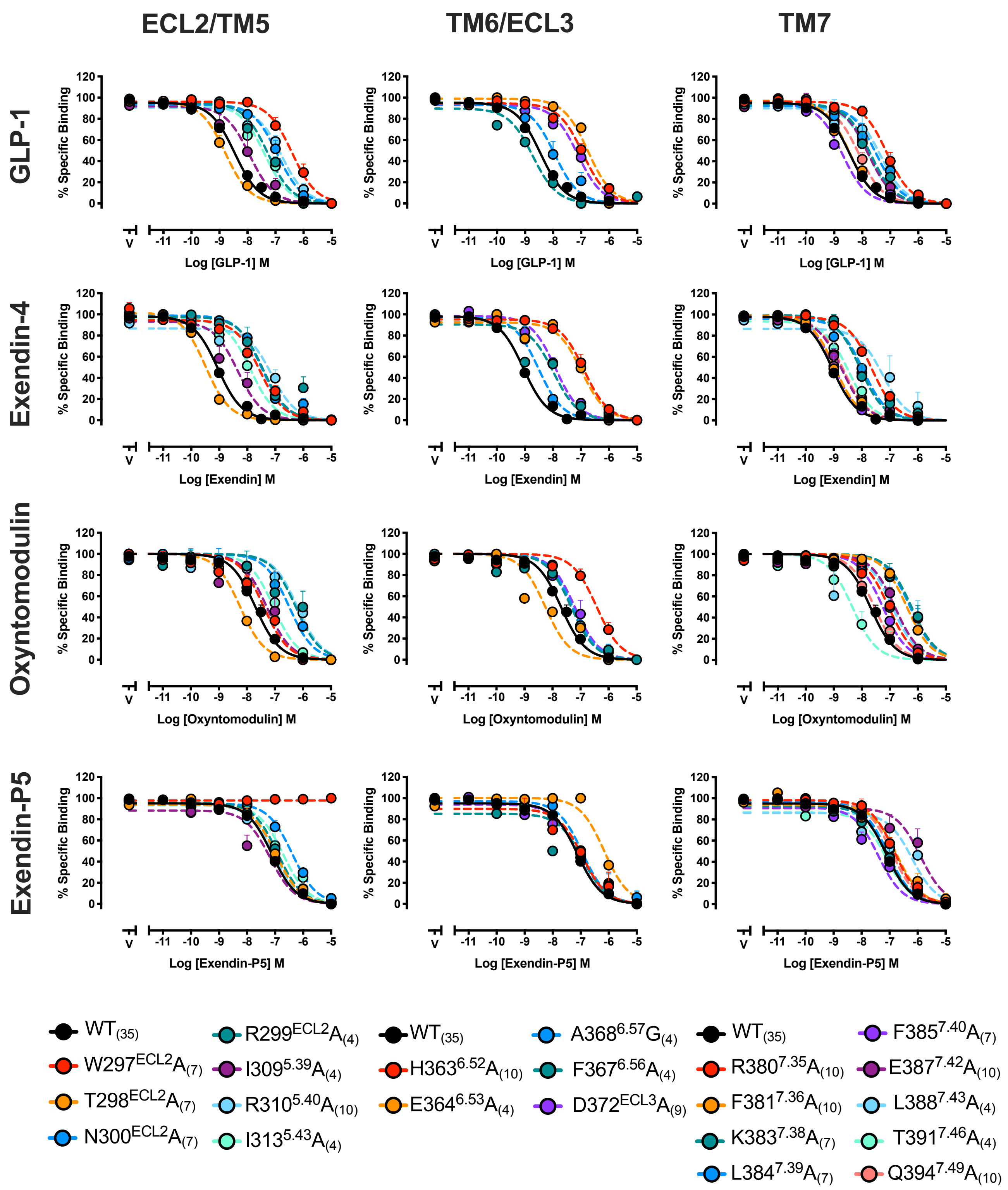

Supplementary Figure 6. Inhibition binding curves for wildtype and alanine mutants of GLP-1R residues within TMs 4-7. Equilibrium competition binding assays assessing the ability of GLP-1, oxyntomodulin, exendin-4 and exendin-P5 to displace the radioligand ^{125}I-exendin(9-39), in Cho-FlpIn cells overexpressing wildtype or mutant GLP-1Rs. Data are presented as % specific binding with 100% binding defined as total probe binding in the absence of competing ligand and non-specific (0%) binding determined as probe binding in the presence of 10 μM exendin(9-39). Data are means + s.e.m. of 4-10 independent experiments for mutant receptors performed in duplicate. For the GLP-1 dataset, the exact no of individual experiments are shown in parenthesis and subscript on the figure for each receptor construct. Source data are provided in the Source Data file.

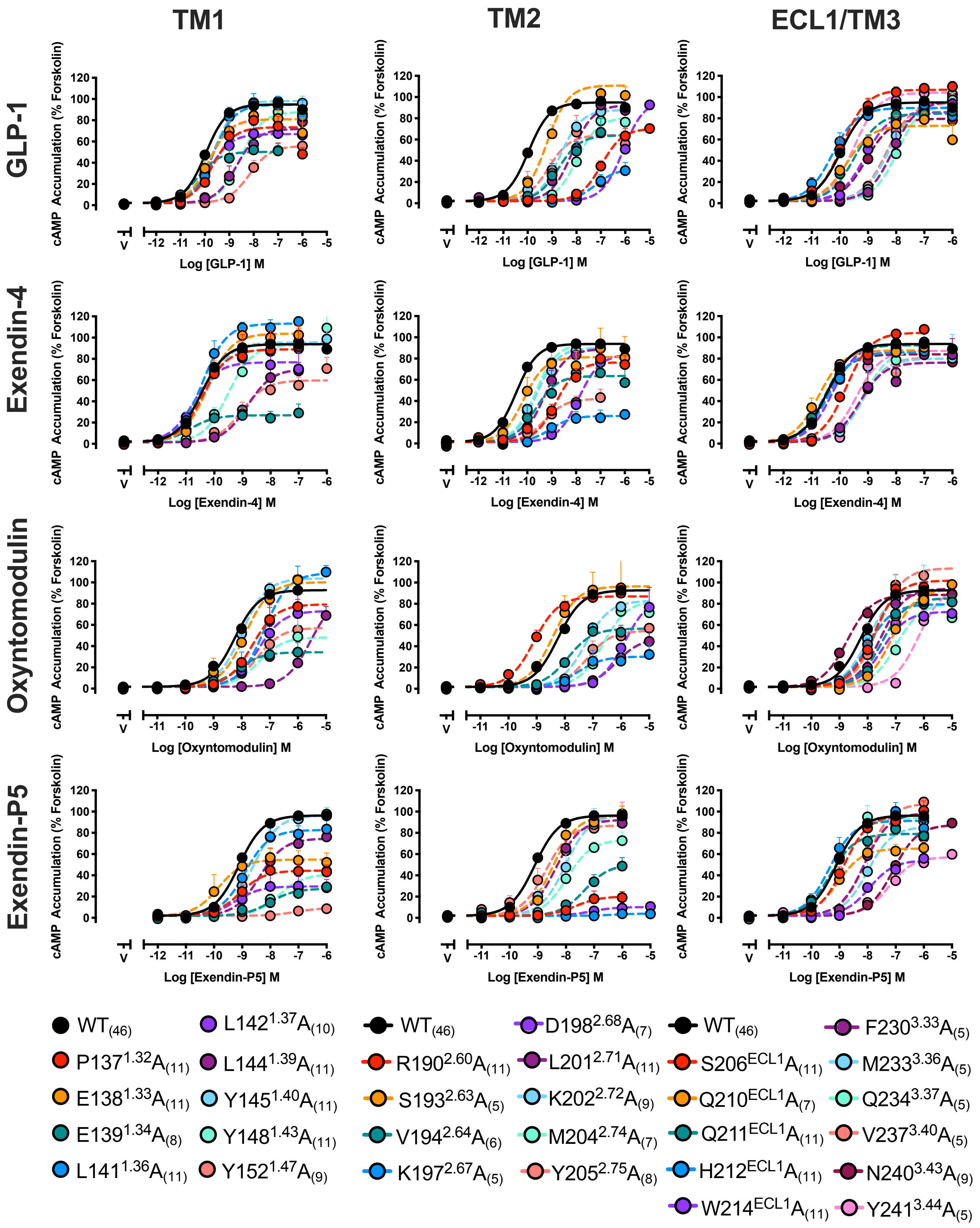

Supplementary Figure 7. cAMP accumulation in ChoFlpIn cells expressing wildtype or alanine mutants of GLP-1R residues within TMs 1-3. Concentration response curves for cAMP production by GLP-1, oxyntomodulin, exendin-4 and exendin-P5 in Cho-FlpIn cells overexpressing wildtype or mutant GLP-1Rs. Data are presented as % cAMP accumulation mediated by 10uM forskolin. Data are means + s.e.m. of 5-11 independent experiments for mutant receptors performed in duplicate. For the GLP-1 dataset, the exact no of individual experiments are shown in parenthesis and subscript on the figure for each receptor construct. Source data are provided in the Source Data file.

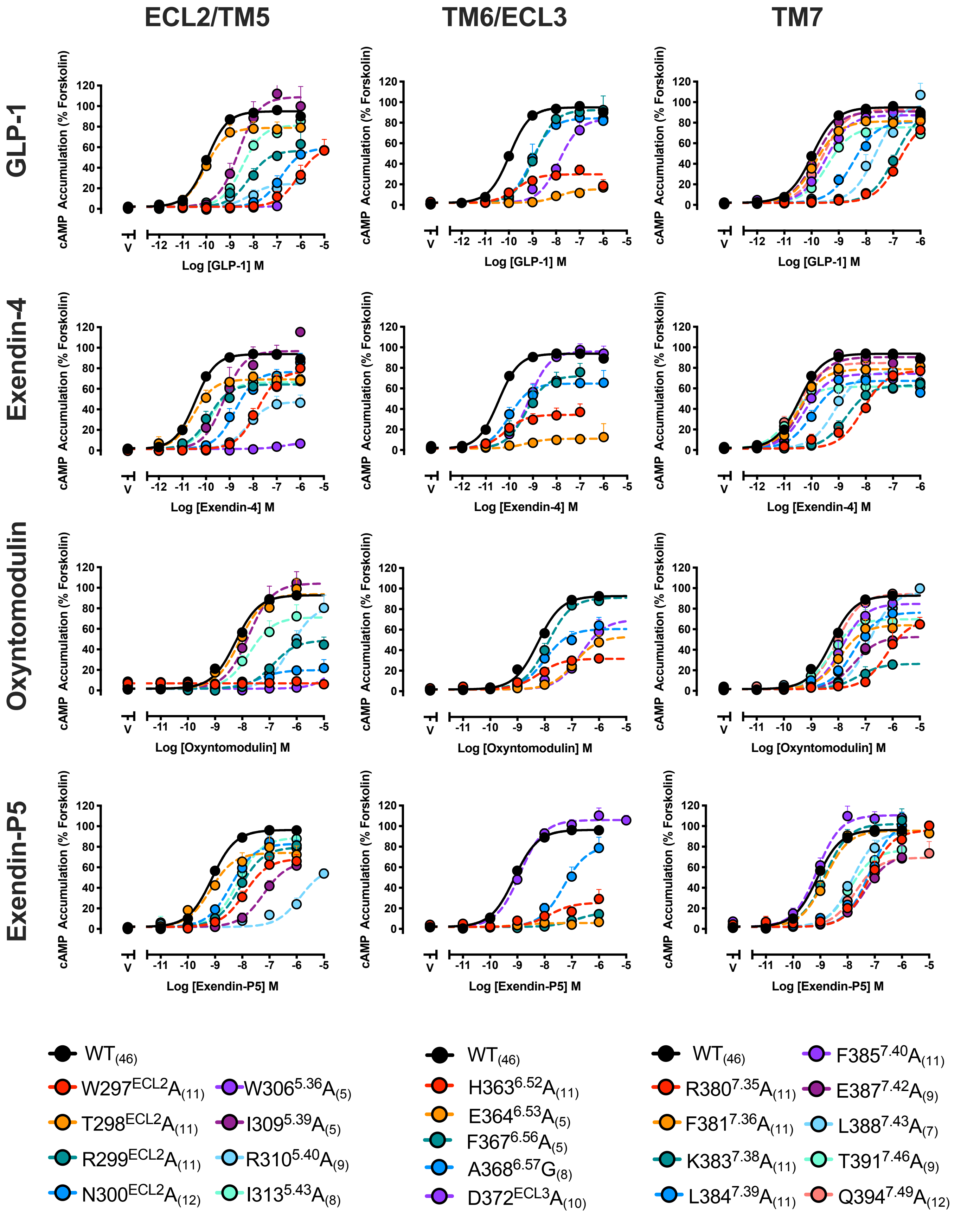

Supplementary Figure 8. cAMP accumulation in ChoFlpIn cells expressing wildtype or alanine mutants of GLP-1R residues within TMs 4-7. Concentration response curves for cAMP production by GLP-1, oxyntomodulin, exendin-4 and exendin-P5 in Cho-FlpIn cells overexpressing wildtype or mutant GLP-1Rs. Data are presented as % cAMP accumulation mediated by 10uM forskolin. Data are means + s.e.m. of 5-12 independent experiments for mutant receptors performed in duplicate. For the GLP-1 dataset, the exact no of individual experiments are shown in parenthesis and subscript on the figure for each receptor construct. Source data are provided in the Source Data file.

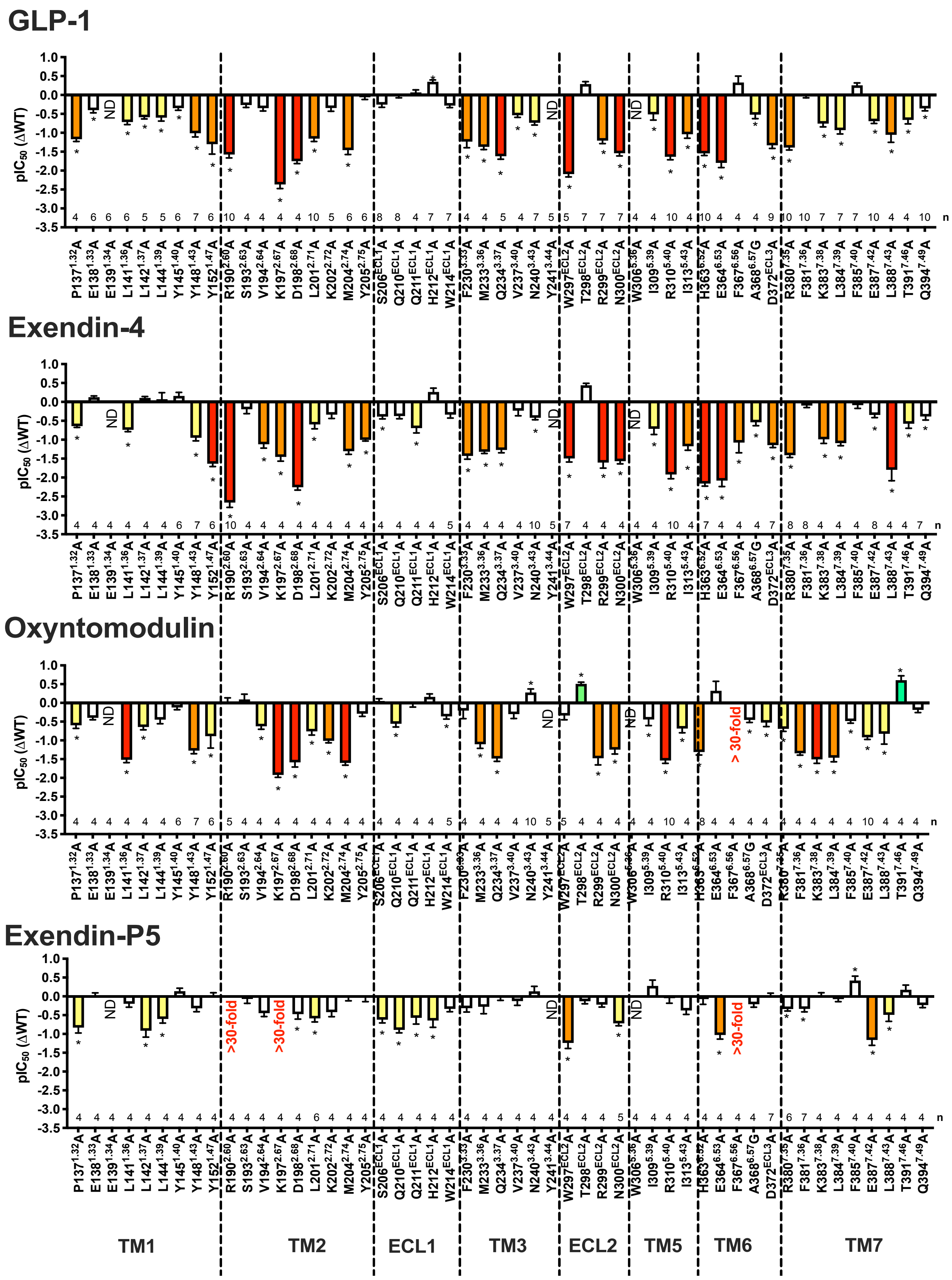

Supplementary Figure 9. Agonist affinity profiles of GLP-1R alanine mutants reveal the importance of individual residues for peptide affinity. pIC50 values for each peptide were derived from radioligand inhibition binding experiments from data in Supplemental Figures 5-6. Bars represent differences in calculated affinity (pIC50) values for each mutant relative to the wild-type receptor for GLP-1, exendin-4, oxyntomodulin and exendin-P5. Statistical significance of changes in affinity in comparison with wild-type was determined by one-way analysis of variance and Dunnett's post-test, and are indicated with an asterisk (*, $p < 0.05$). Data that are statistically significant are coloured based on the extent of effect. All values are derived from fitting inhibition binding curves to grouped data (curve fitting is shown on the grouped data in Supplementary Figures 5-6) to derive a mean + s.e.m. The number of independent experiments that were performed to obtain the grouped data is shown for each receptor construct on the figure (n). ND. Not determined. Linked to Figures 4-5.

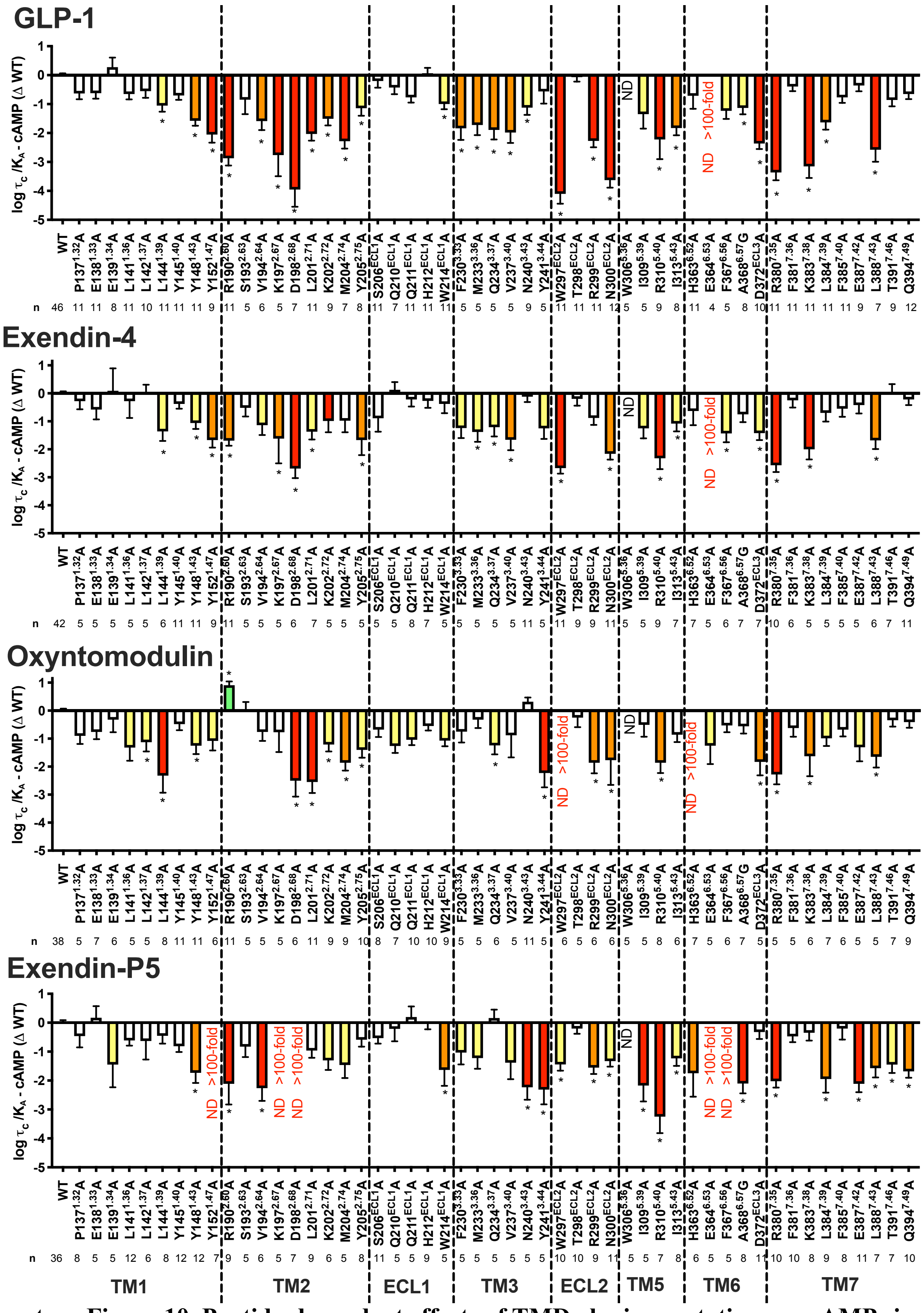

Supplementary Figure 10. Peptide-dependent effects of TMD alanine mutations on cAMP signalling. Differences in the coupling efficiency (log(τ/KA)c) for cAMP formation of TMD mutations compared to the wild-type receptor by GLP-1, exendin-4, oxyntomodulin and exendin-P5 were determined by applying the operational model of agonism to concentration response data shown in supplemental figures 7-8. Statistical significance of changes in coupling efficacy was determined by one-way analysis of variance and Dunnett's post-test, and those of significance are indicated with an asterisk (*, $p < 0.05$ compared with wild-type). Data that are statistically significant are coloured based on the direction and extent of effect. All values were derived from fitting the operational model of agonism to grouped data (curve fitting is shown on the grouped data in Supplementary Figures 7-8) to derive a mean + s.e.m. The number of independent experiments that were performed to obtain the grouped data is shown for each receptor construct on the figure (n). ND. Not determined. Linked to Figures 4-5.

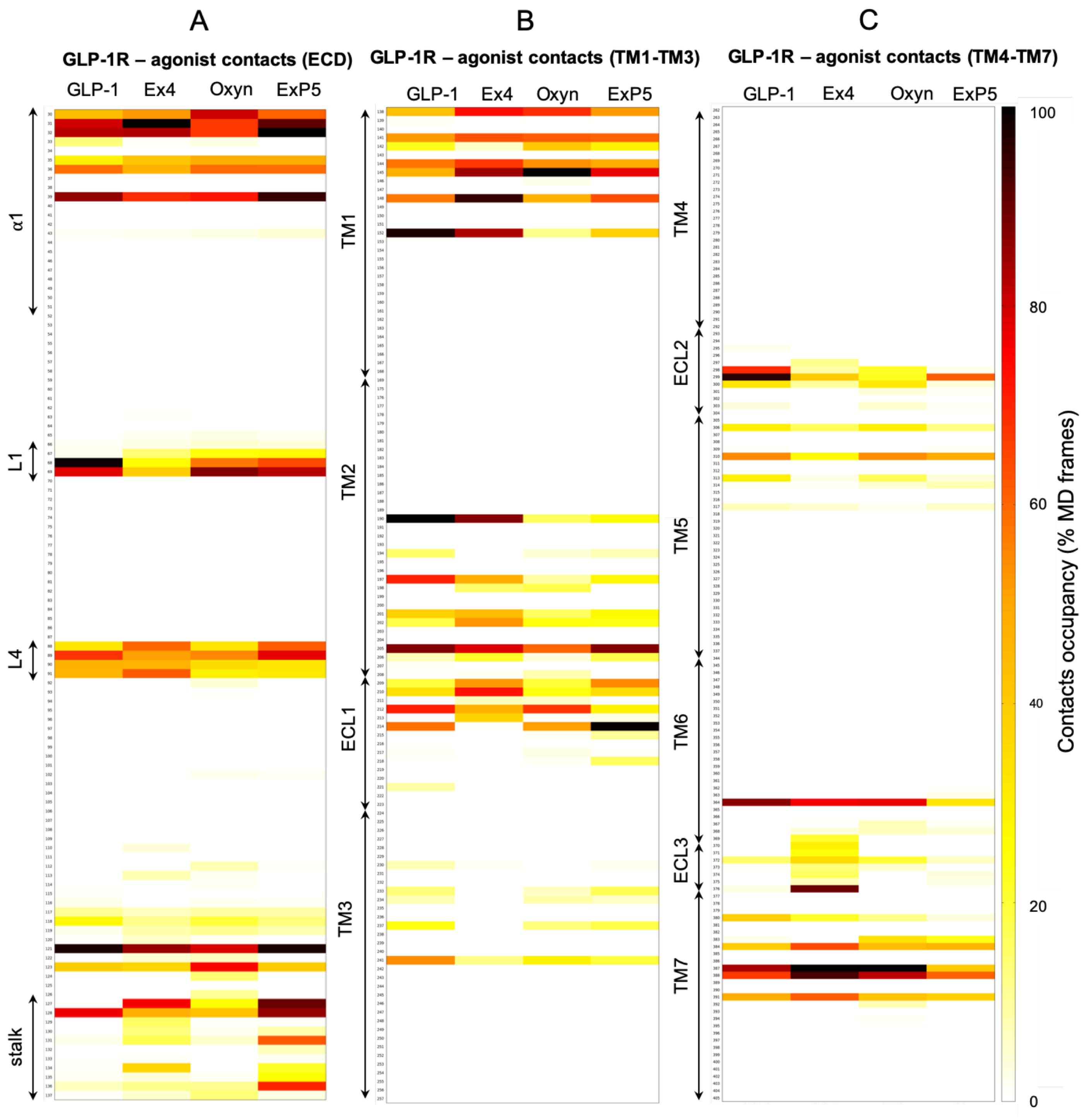

Supplementary Figure 11. Heatmap showing the GLP-1R contacts and peptide agonists from MD simulations. The GLP-1R primary sequence is divided into three segments: ECD (A), TM1 to TM3 (B), and TM4 to TM7 (C). ICL1, ICL2, and IC3 have been omitted as they are not involved in interactions. For each agonist (GLP-1, exendin-4 (Ex4), oxyntomodulin (Oxyn), exendin-P5 (ExP5)), data are reported as the percentage of MD frames (occupancy) with at least one interatomic contact and normalized for the maximum occupancy. Colours are scaled according to the occupancy with black indicating 100 % occupancy, white 0% occupancy and yellow (low) through to red (high) occupancies in between, as per the contacts occupancy scale bar.

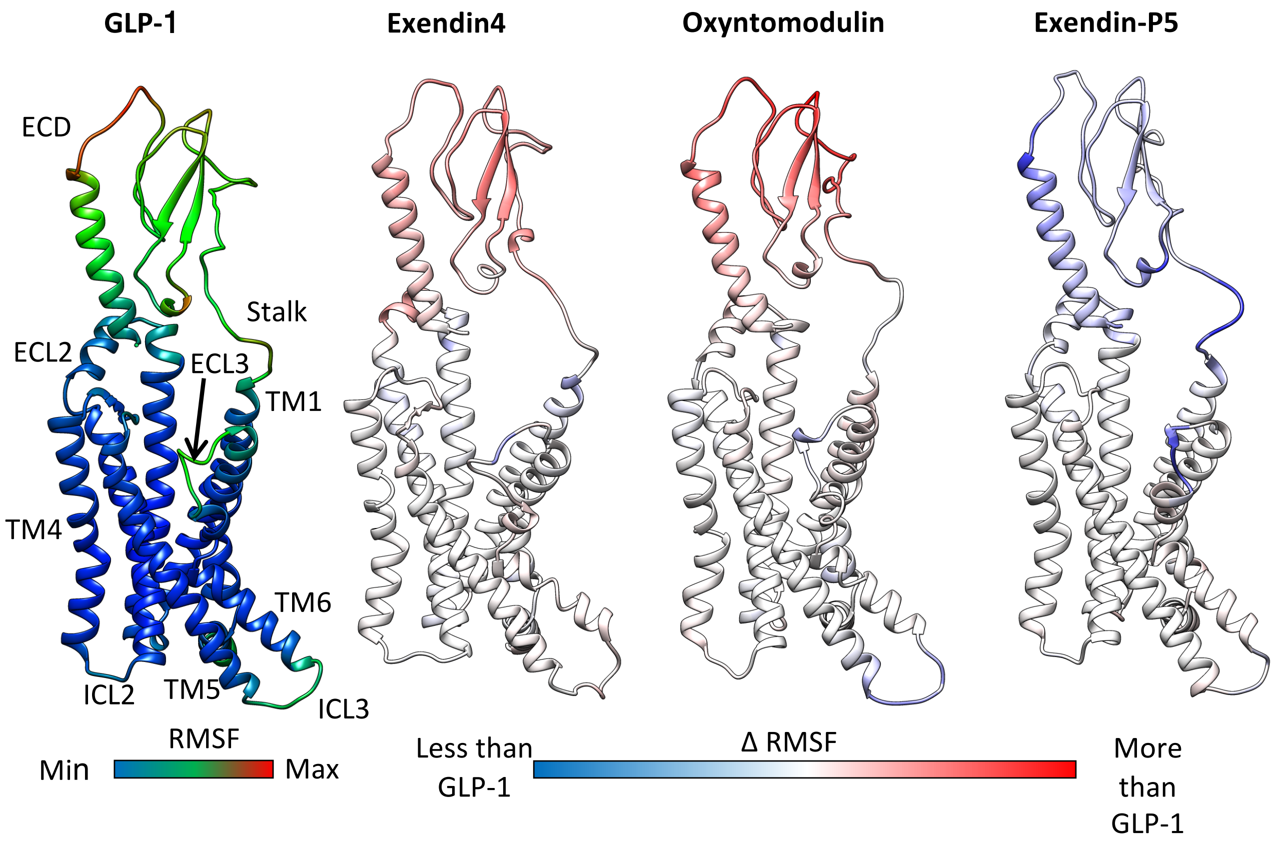

Supplementary Figure 12. Comparison of the GLP1-R RMSF observed in MD simulations between the different GLP-1R:agonist complexes. For each residue, the RMSF in the presence of GLP-1 (left, where low RMSF is blue and high RMSF is red) is subtracted from the RMSF computed in the presence of the other agonists. Values are plotted on the backbone (ribbon) of the receptor for these three ligands with blue indicating less RMSF, white the same RMDF and red a greater RMSF during the simulation compared to GLP-1.

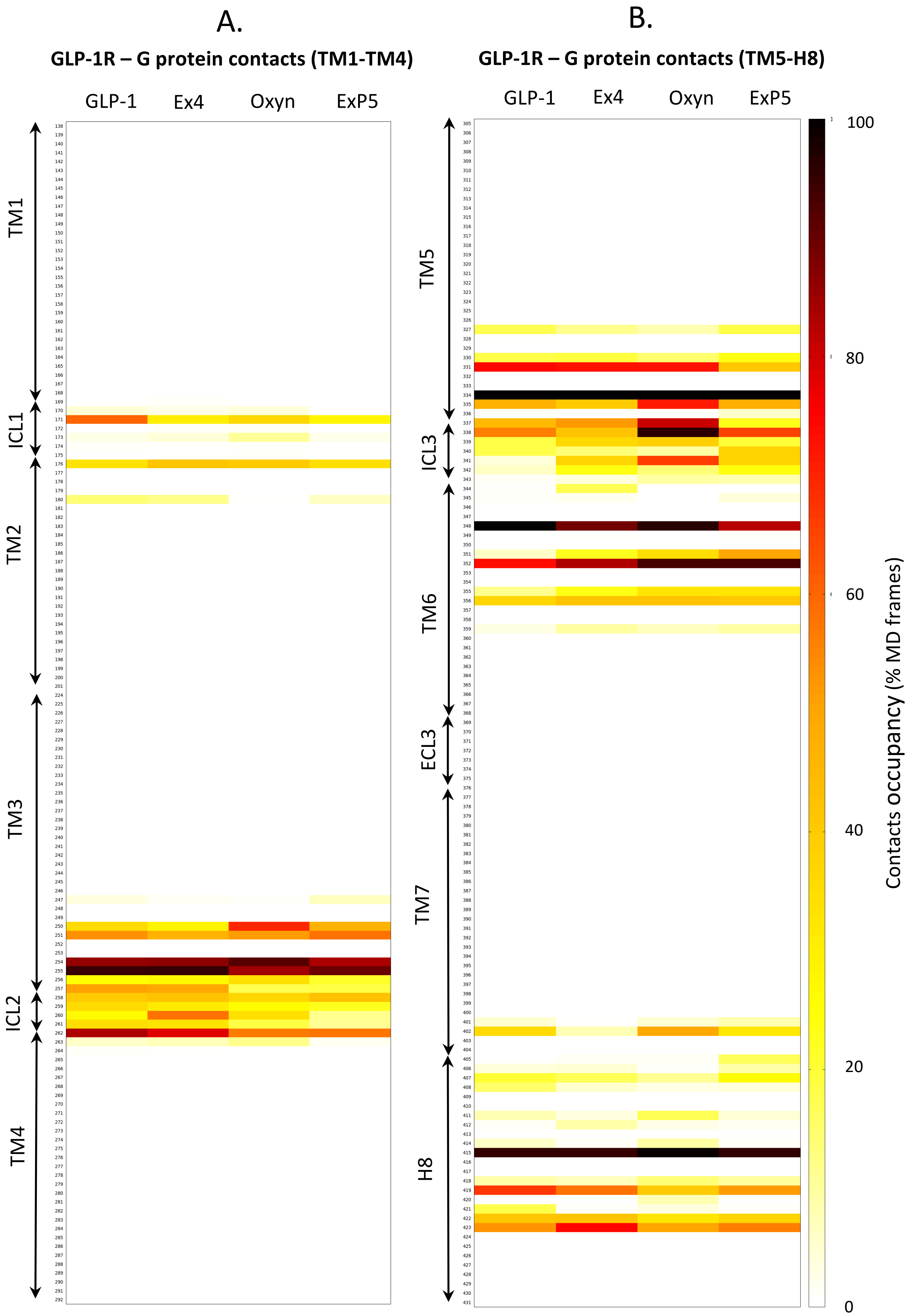

Supplementary Figure 13. Heatmap showing the GLP-1R contacts with Gs in the presence of GLP-1, exendin-4, oxyntomodulin and exendin-P5. The GLP-1R primary sequence is divided into two segments: TM1 to TM4 (A), and TM5 to H8 (B). ECD, ECL1, and ECL2 have been omitted as these do not contact Gs. For each agonist (GLP-1, exendin-4 (Ex4), oxyntomodulin (Oxyn), exendin-P5 (ExP5)), data are reported as the percentage of MD frames (occupancy) with at least one interatomic contact and normalised for the maximum occupancy, where yellow indicates low occupany and red high occupancy as per the contacts occupancy scale bar.

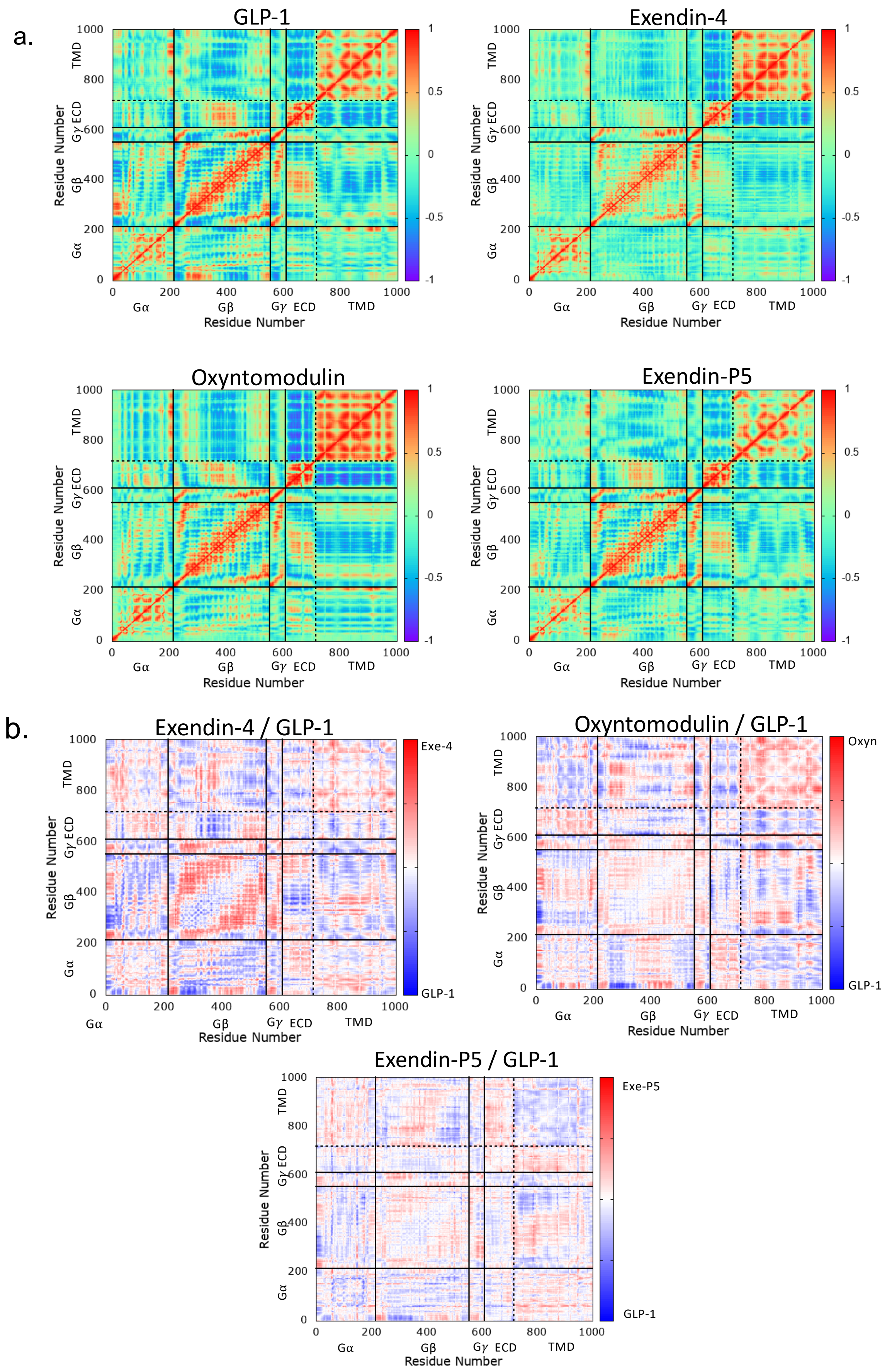

Supplementary Figure 14. Correlation of motion between residues during MD simulations. Sequences corresponding to Gs subunits a, b, and g are delimited by solid lines. GLP-1R ECD and TMD are divided by a dashed line. a, Motions correlated are indicated in orange or red, while those anti-correlated are in blue. b, The correlation matrix of GLP-1:GLP-1R:Gs was subtracted from the correlation matrix of exendin-4:GLP-1R:Gs, oxyntomodulin:GLP-1R:Gs, or exendin-P5:GLP-1R:Gs, respectively. Red indicates greater positive correlations in the presence of exendin-4, oxyntomodulin or exendin-P5 systems; blue shows greater positive correlations in the presence of GLP-1.

Supplementary Figure 15.

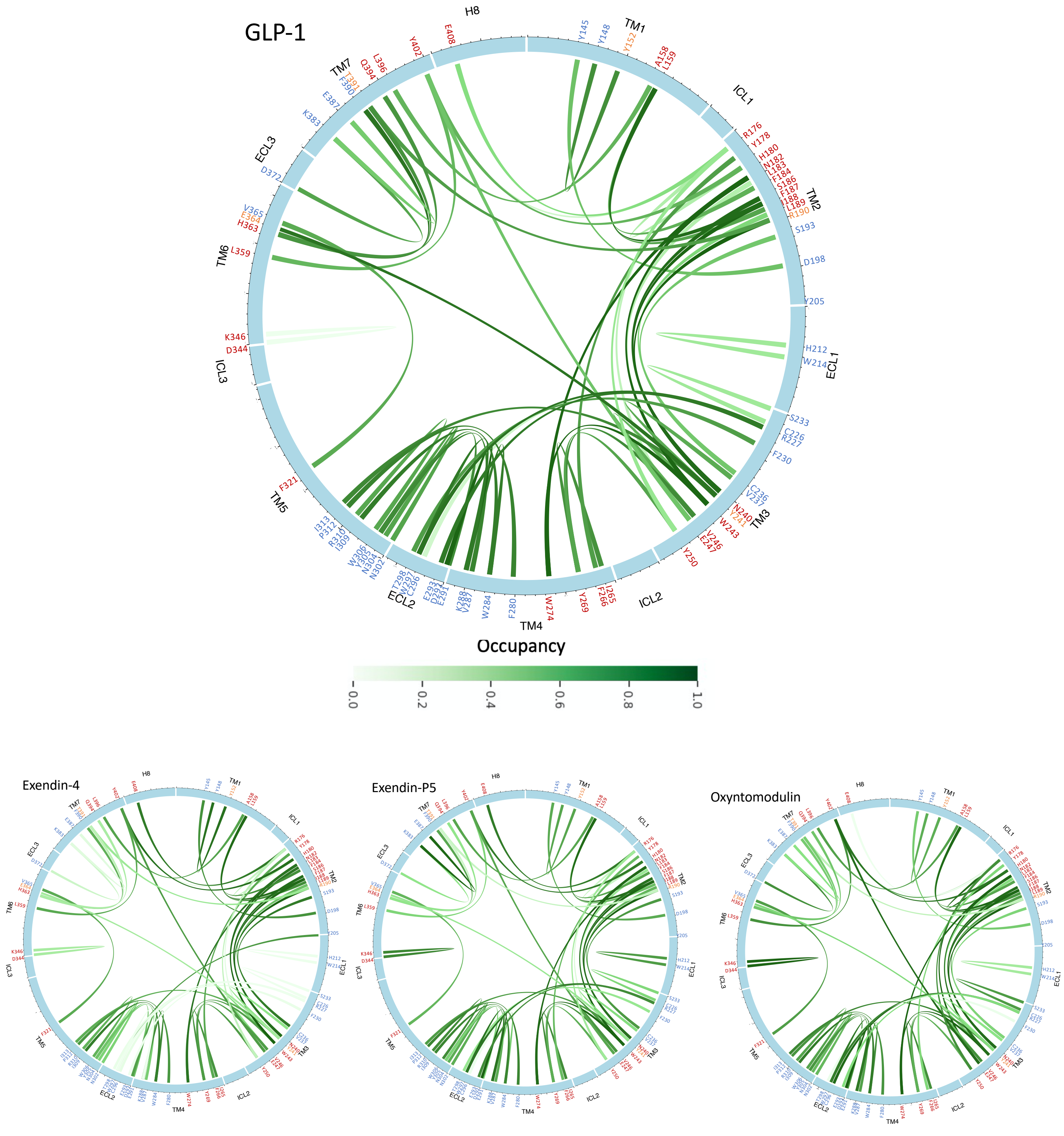

Supplementary Figure 15. Cluster analysis of the contacts between GLP-1R side chains for the four peptide-receptor-Gs complexes. Flare plots showing receptor-receptorcontacts within the GLP-1R TM bundle when occupied by each peptide. Only interactions that have occupancy during the course of MD simulation in at least one of the complexes are shown. The intensity of green colouring is scaled to the degree of occupancy for each interaction. The reference GLP-1 is shown on the left with comparator plots for oxyntomodulin, exendin-4 and exendin-P5 on the right. Residue labels are coloured according to their location in the TM bundle; blue - located on the extracellular half (or within the TM binding cavity) of the TM bundle; orange - line the bottom of the peptide binding cavity; red - located in the intracellular half of the TM bundle, below the peptide binding cavity.

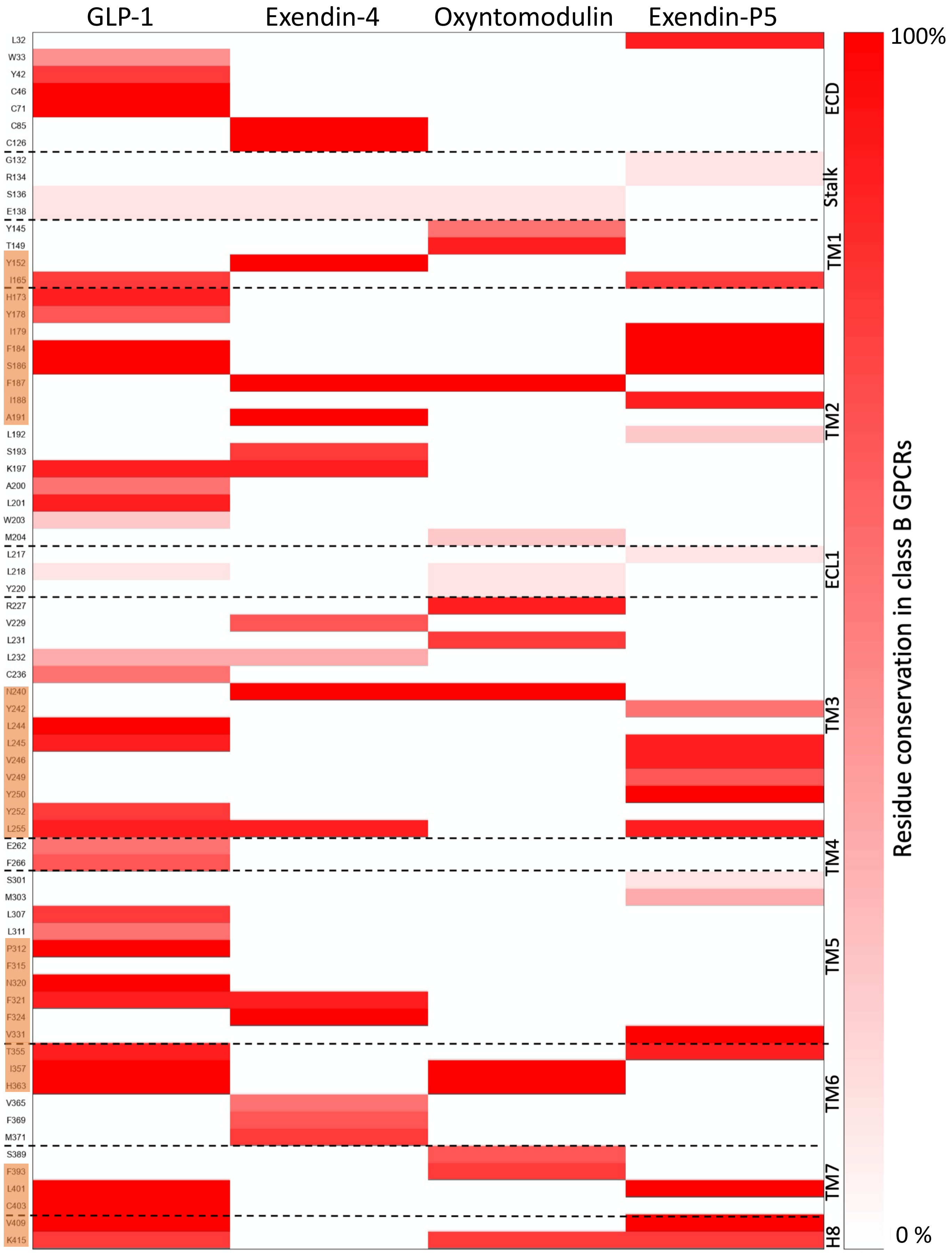

Supplementary Figure 16. Comparison between residues critical for communication between communities of the GLP-1R, when in complex with different peptides, and Gs. Edge residues ordered according to the position on GLP-1R sequence. The intensity of the red colouring is scaled according to conservation score of the residue across the class B1 GPCRs, calculated according to Consurf analysis70. Residue labels highlighted in orange are located in the intracellular half of the TM bundle, below the peptide binding cavity.

Supplementary Table 1. Interactions between GLP-1R and GLP-1 (Zhang, et al. 2020), oxyntomodulin, exendin-4 and ExP5 (Liang, et al. 2018). Receptor TMD residues within 4 Å of the exendin-4 and oxyntomodulin within the static cryo-EM structures are detailed.

GLP-1R:GLP-1:Gs		GLP-1R:Oxyn:Gs		GLP-1R:Ex4:Gs		GLP-1R:ExP5:Gs	
GLP-1R	**GLP-1**	**GLP-1R**	**Oxy**	**GLP-1R**	**Ex4**	**GLP-1R**	**ExP5**
V237$^{3.40}$	H7	V237$^{3.40}$	H1	V237$^{3.40}$	H1	V237$^{3.40}$	L2
W306$^{5.36}$	H7	W306$^{5.36}$	H1				
I309$^{5.39}$	H7						
R310$^{5.40}$	H7 (water mediated)	R310$^{5.40}$	H1			R310$^{5.40}$	E1
I313$^{5.43}$	H7					I313$^{5.43}$	L2
Q234$^{3.37}$	H7	Q234$^{3.37}$	H1				
		Y241$^{3.44}$	H1			A368$^{6.57}$	E1
Y241$^{3.44}$	A8 (water mediated)						
L384$^{7.39}$	A8	L384$^{7.39}$	S2			L384$^{7.39}$	V3
E387$^{7.42}$	A8	E387$^{7.42}$	S2			E387$^{7.42}$	V3
L388$^{7.43}$	A8	L388$^{7.43}$	S2			L388$^{7.43}$	V3
Y152$^{1.47}$	E9	Y152$^{1.47}$	Q3	Y152$^{1.47}$	E3	Y152$^{1.47}$	D4
R190$^{2.60}$	E9	V194$^{2.64}$	Q3	R190$^{2.60}$	E3	V194$^{2.64}$	D4
Y241$^{3.44}$	E9 (water mediated)	Y148$^{1.43}$	Q3	Y148$^{1.43}$	E3	K197$^{2.67}$	D4
L388$^{7.43}$	E9 (water mediated)	L388$^{7.43}$	Q3			M233$^{3.36}$	D4
T298^{ECL2}	G10 (water mediated)						
N300^{ECL2}	G10						
W306$^{5.36}$	G10	W306$^{5.36}$	G4	W306$^{5.36}$	G4		
W306$^{5.36}$	T11	W306$^{5.36}$	T5			W306$^{5.36}$	N5
D372^{ECL3}	T11					Q234$^{3.37}$	N5
R380$^{7.35}$	T11						
L384$^{7.39}$	T11						
L141$^{1.36}$	F12	L141$^{1.36}$	F6	L141$^{1.36}$	F6		
L144$^{1.39}$	F12	L144$^{1.39}$	F6	L144$^{1.39}$	F6		
Y148$^{1.43}$	F12					L384$^{7.39}$	V7
L388$^{7.43}$	F12					L388$^{7.43}$	V7
K197$^{2.67}$	T13	K197$^{2.67}$	T7	K197$^{2.67}$	T7		
F230$^{3.33}$	T13						
M233$^{3.36}$	T13			M233$^{3.36}$	T7		
T298^{ECL2}	T13	T298^{ECL2}	T7				
T298^{ECL2}	S14	T298^{ECL2}	S8	T298^{ECL2}	S8		
R299^{ECL2}	S14						
N300^{ECL2}	S14	N300^{ECL2}	S8				
R380$^{7.35}$	D15	R380$^{7.35}$	D9			R380$^{7.35}$	D10
L384$^{7.39}$	D15						
L201$^{2.71}$	V16	L201$^{2.71}$	Y10			L141$^{1.36}$	L11
Y205^{ECL1}	S17					L201$^{2.71}$	S12
T298^{ECL2}	S17	T298^{ECL2}	S11	T298^{ECL2}	S11	R298^{ECL2}	S12
R299^{ECL2}	S17	R299^{ECL2}	S11	R299^{ECL2}	S11		

$R299^{ECL2}$	S18	$R299^{ECL2}$	K12			$R299^{ECL2}$	K13
$P137^{1.32}$	Y19						
$E138^{1.33}$	Y19	$E138^{1.33}$	Y13			$E138^{1.33}$	Q14
		$L141^{1.36}$	Y13			$L141^{1.36}$	Q14
$Y205^{ECL1}$	L20	$Y205^{ECL1}$	L14			$Y205^{ECL1}$	M15
						$L201^{2.71}$	M15
						$K202^{2.72}$	M15
						$S206^{ECL1}$	M15
$V30^{ECD}$	E21						
$S31^{ECD}$	E21						
$L32^{ECD}$	E21					$L32^{ECD}$	E16
$Y205^{ECL1}$	E21	$Y205^{ECL1}$	D15	$Y205^{ECL1}$	E15	$Y205^{ECL1}$	E16
$Q221^{ECL1}$	E21						
$R299^{ECL2}$	E21	$R299^{ECL2}$	D15			$R299^{ECL2}$	E16
$L32^{ECD}$	A24						
$Q210^{ECL1}$	A24	$Y205^{ECL1}$	R18			$Y205^{ECL1}$	A19
						$L32^{ECD}$	V20
						$P90^{ECD}$	V20
$E128^{ECD}$	K26						
$Q210^{ECL1}$	E27						
$L32^{ECD}$	F28						
$T35^{ECD}$	F28						
$W39^{ECD}$	F28					$W39^{ECD}$	F23
$W214^{ECL1}$	F28			$W214^{ECL1}$	F22		F23
$T35^{ECD}$	I29					$V20^{ECD}$	I24
$Y69^{ECD}$	I29					$Y69^{ECD}$	I24
$P90^{ECD}$	I29					$P90^{ECD}$	I24
$W91^{ECD}$	I29					$W91^{ECD}$	I24
$W214^{ECL1}$	W31			$W214^{ECL1}$	W25	$W214^{ECL1}$	W26
						$H212^{ECL1}$	W26
$W39^{ECD}$	L32						
$E68^{ECD}$	L32						
$Y69^{ECD}$	L32						
$Y69^{ECD}$	V33						
$R121^{ECD}$	V33						
$W39^{ECD}$	R36						
$E68^{ECD}$	R36						

Supplementary Table 2. The effects of TMD mutations on peptide affinity and cAMP signalling efficiency. Mutant and WT GLP-1Rs were stably expressed in ChoFlpIn cells and competition inhibition binding curves **(Supplementary Figures 5-6)** and cAMP accumulation concentration response curves (Supplementary Figures 7-8) were generated for each construct for the four agonists. Binding data were analyzed using a three-parameter logistic equation to determine pIC_{50} values, which represent the negative logarithm of the concentration of ligand that inhibits binding of half the total concentration of radiolabelled antagonist, ^{125}I-exendin(9-39). Cell surface expression was determined through antibody detection of the N-terminal c-myc epitope label, with data expressed as a maximum of wildtype human GLP-1R expression. cAMP data were analysed with an operational model of agonism to determine $\log\tau/K_A$ values, which were then corrected to cell surface expression data. Values are expressed as mean ± s.e.m. Data were analysed with one-way analysis of variance and Dunnett's post test (* $p < 0.05$). ND means data that were unable to be experimentally defined. These data are also shown graphically in Supplemental Figures 9-10.

Residue location in the GLP-1R	Construct	Cell surface expression (% WT)	GLP-1		Exendin-4		Oxyntomodulin		Exendin-P5	
			Binding pIC_{50}	cAMP $Log(\tau/K_A)_c$	Binding pIC_{50}	cAMP $Log(\tau/K_A)_c$	Binding pIC_{50}	cAMP $Log(\tau/K_A)_c$	Binding pIC_{50}	cAMP $Log(\tau/K_A)_c$
	**WT**	100±1	8.56±0.03	10.07±0.06	9.05±0.03	10.55±0.07	7.73±0.04	8.34±0.07	7.19±0.05	9.23±0.09
**TM1**	**P137$^{1.32}$A**	102±14	7.39±0.60*	9.44±0.20	8.41±0.03	10.27±0.29	7.13±0.08*	7.44±0.29	6.36±0.14*	8.77±0.39
	**E138$^{1.33}$A**	164±13*	8.16±0.07*	9.45±0.19	9.18±0.04*	9.97±0.35	7.34±0.05	7.58±0.26	7.22±0.06	9.40±0.40
	**E139$^{1.34}$A**	37±8*	ND	10.35±0.33	ND	10.64±0.80	ND	8.02±0.45	ND	7.78±0.78
	**L141$^{1.36}$A**	136±8	7.85±0.07*	9.42±0.19	8.31±0.05*	10.27±0.60	6.22±0.08*	7.02±0.47	6.99±0.09	8.62±0.18
	**L142$^{1.37}$A**	94±11	7.97±0.05*	9.52±0.23	9.16±0.04	10.57±0.29	7.09±0.07*	7.21±0.33	6.27±0.17*	8.60±0.64
	**L144$^{1.39}$A**	25±6*	7.96±0.09*	9.02±0.21*	9.13±0.17	9.19±0.34*	7.29±0.11	6.02±0.61*	6.59±0.11*	8.78±0.27
	**Y145$^{1.40}$A**	88±8	8.21±0.06	9.37±0.15	9.21±0.09	10.17±0.17	7.61±0.06	7.86±0.22	7.33±0.08	8.42±0.20
	**Y148$^{1.43}$A**	67±8	7.55±0.10*	8.49±0.17*	8.11±0.08*	9.49±0.21*	6.46±0.08*	7.09±0.30*	6.87±0.09	7.50±0.35*
	**Y152$^{1.47}$A**	71±4	7.26±0.26*	8.02±0.28*	7.42±0.07*	8.88±0.27*	6.85±0.32*	7.26±0.34	7.19±0.09	ND
**TM2**	**R190$^{2.60}$A**	53±3	6.99±0.09*	7.20±0.25*	6.39±0.13*	8.86±0.18*	7.77±0.10	9.25±0.13*	ND	7.12±0.72*
	**S193$^{2.63}$A**	93±7	8.30±0.06	9.22±0.50	8.86±0.12	10.03±0.31	7.83±0.14	8.36±0.29	7.12±0.11	8.40±0.36
	**V194$^{2.64}$A**	95±7	8.21±0.07	8.48±0.31*	7.93±0.10*	9.41±0.35	7.11±0.08*	7.58±0.32	6.74±0.09	6.97±0.44*
	**K197$^{2.67}$A**	27±3*	6.20±0.11*	7.31±0.73*	7.60±0.11*	8.93±0.88*	5.81±0.07*	7.57±0.71	ND	ND
	**D198$^{2.68}$A**	74±2	6.81±0.06*	6.12±0.60*	6.79±0.07*	7.86±0.34*	6.15±0.13*	5.84±0.57*	6.72±0.13*	ND
	**L201$^{2.71}$A**	94±2	7.41±0.07*	8.04±0.23*	8.46±0.12*	9.18±0.28*	6.97±0.10*	5.79±0.39*	6.60±0.09*	8.27±0.25
	**K202$^{2.72}$A**	94±3	8.22±0.08	8.56±0.23*	8.72±0.10	9.56±0.40	6.72±0.05*	7.13±0.25*	6.77±0.12	7.93±0.33
	**M204$^{2.74}$A**	99±2	7.10±0.11*	7.79±0.26*	7.75±0.08*	9.57±0.41	6.13±0.06*	6.48±0.28*	7.17±0.10	7.77±0.45
	**Y205$^{2.75}$A**	68±5*	8.51±0.07	8.93±0.26*	8.05±0.03*	8.88±0.54*	7.44±0.07	6.94±0.28*	7.16±0.12	8.64±0.24
**ECL1**	**S206^{ECL1}A**	111±6	8.31±0.07	9.87±0.23	8.66±0.06*	9.66±0.48	7.78±0.06	7.66±0.26	6.57±0.09*	8.70±0.19
	**Q210^{ECL1}A**	88±4	8.55±0.07	9.64±0.23	8.68±0.07	10.68±0.27	7.18±0.09*	7.08±0.25*	6.30±0.08*	9.02±0.43
	**Q211^{ECL1}A**	112±5	8.63±0.07	9.30±0.18	8.36±0.13*	10.33±0.25	7.71±0.09	7.30±0.19	6.62±0.17*	9.43±0.36
	**H212^{ECL1}A**	103±3	8.91±0.05*	10.13±0.19	9.32±0.10	10.28±0.25	7.89±0.08	7.79±0.16	6.55±0.18*	9.21±0.21

	**W214^{ECL1}A**	99±5	8.28±0.06	9.07±0.18*	8.72±0.09	10.17±0.33	7.37±0.07*	7.27±0.20	6.86±0.08	7.60±0.55
**TM3**	**F230$^{3.33}$A**	69±7*	7.33±0.16*	8.23±0.40*	7.62±0.09*	9.31±0.36	7.53±0.21	7.59±0.39	6.87±0.10	8.20±0.41
	**M233$^{3.36}$A**	70±5	7.19±0.07*	8.35±0.35*	7.73±0.05*	9.17±0.35*	6.63±0.11*	8.02±0.30	6.91±0.18	8.01±0.37
	**Q234$^{3.37}$A**	46±4*	6.94±0.08*	8.18±0.33*	7.78±0.08*	9.34±0.33	6.25±0.07*	7.10±0.32	7.18±0.10	9.39±0.29
	**V237$^{3.40}$A**	141±3*	8.02±0.05	8.09±0.36*	8.82±0.13	8.89±0.37*	7.43±0.12	7.46±0.79	7.06±0.11	7.85±0.57
	**N240$^{3.43}$A**	87±3	7.83±0.06*	8.95±0.25*	8.63±0.05*	10.43±0.19	8.01±0.09*	8.66±0.15	7.32±0.13	7.00±0.43*
	**Y2413.44A**	95±2	ND	9.51±0.42	ND	9.30±0.38	ND	6.11±0.51*	ND	6.92±0.51*
**ECL2**	**W297^{ECL2}A**	60±6	6.47±0.08*	5.97±0.34*	7.56±0.09*	7.88±0.20*	7.39±0.11	ND	5.95±0.15*	7.79±0.22*
	**T298^{ECL2}A**	85±6	8.85±0.06	10.01±0.16	9.49±0.06	10.35±0.24	8.24±0.05*	8.09±0.34	7.06±0.07	9.03±0.18
	**R299ECL2A**	94±5	7.35±0.08*	7.80±0.22*	7.45±0.15*	9.67±0.25	6.26±0.18*	6.48±0.38*	6.97±0.06	7.68±0.22*
	**N300^{ECL2}A**	130±6	7.02±0.07*	6.44±0.26*	7.49±0.07*	8.39±0.21*	6.49±0.12*	6.57±0.88*	6.47±0.06*	7.90±0.19*
**TM5**	**W306$^{5.36}$A**	ND	ND	ND	ND	ND	ND	ND	ND	ND
	**I309$^{5.39}$A**	75±9	8.05±0.15*	8.72±0.50	8.34±0.15*	9.30±0.36	7.29±0.17*	7.83±0.42	7.47±0.15	7.06±0.55*
	**R310$^{5.40}$A**	38±1*	6.93±0.08*	7.85±0.68*	7.13±0.11*	8.23±0.39*	6.20±0.07*	6.48±0.37*	7.14±0.14	5.98±0.57*
	**I313$^{5.43}$A**	106±5	7.52±0.11*	8.24±0.25*	7.88±0.11*	9.47±0.28*	7.05±0.12*	7.48±0.26	6.81±0.11	8.00±0.26*
**TM6**	**H363$^{6.52}$A**	59±4*	7.02±0.06*	9.36±0.45	6.89±0.07*	9.92±0.51	6.42±0.08*	ND	7.11±0.14	7.48±0.81*
	**E364$^{6.53}$A**	41±4	6.77±0.13*	ND	6.97±0.16*	ND	8.06±0.25	7.09±0.66	6.16±0.11*	ND
	**F367$^{6.56}$A**	ND	8.89±0.17	8.84±0.28	7.98±0.27*	9.12±0.32*	ND	7.81±0.24	ND	ND
	**A368$^{6.57}$G**	85±6	8.04±0.11*	8.94±0.22*	8.51±0.09*	9.80±0.28	7.28±0.07*	7.78±0.26	6.98±0.08	7.13±0.34*
**ECL3**	**D372^{ECL3}A**	86±4	7.23±0.08*	7.71±0.19*	7.91±0.06*	9.13±0.25*	7.21±0.11*	6.51±0.48*	7.19±0.08	8.91±0.24
**TM7**	**R380$^{7.35}$A**	73±5*	7.17±0.06*	6.71±0.27*	7.64±0.06*	7.98±0.24*	7.04±0.07*	6.06±0.35*	6.85±0.05*	7.21±0.22*
	**F381$^{7.36}$A**	92±5	8.54±0.05	9.68±0.16	8.96±0.05	10.30±0.26	6.39±0.05*	7.72±0.31	6.85±0.08*	8.76±0.20
	**K383$^{7.38}$A**	78±4*	7.81±0.09*	6.92±0.40*	8.06±0.11*	8.55±0.36*	6.23±0.10*	6.71±0.71*	7.19±0.09	8.88±0.27
	**L384$^{7.39}$A**	74±7*	7.63±0.10*	8.44±0.25*	7.96±0.07*	9.85±0.31	6.27±0.11*	7.35±0.27	7.11±0.07	7.28±0.47*
	**F385$^{7.40}$A**	110±7	8.82±0.06	9.30±0.19	8.97±0.09	10.00±0.29	7.25±0.06*	7.66±0.24	7.61±0.12*	9.03±0.38
	**E387$^{7.42}$A**	104±6	7.87±0.06*	9.71±0.20	8.71±0.07*	10.13±0.30	6.82±0.06*	7.03±0.50	6.03±0.14*	7.12±0.29*
	**L388$^{7.43}$A**	116±9	7.51±0.20*	7.49±0.41*	7.26±0.29*	8.87±0.31*	6.91±0.27*	6.69±0.38*	6.69±0.17*	7.66±0.32*
	**T391$^{7.46}$A**	86±6	7.90±0.10*	9.21±0.22	8.47±0.12*	10.58±0.30	8.34±0.12*	8.00±0.23	7.36±0.13	7.78±0.29*
	**Q394$^{7.49}$A**	103±3	8.20±0.06*	9.42±0.18	8.67±0.09*	10.33±0.20	7.55±0.07	7.93±0.20	6.96±0.07	7.55±0.22*

note: Data in black were generated for this paper
Data in red are from Wootten et al., PNAS, 2013
Data in green are from Lei et al., JBC, 2018
Data in purple are from Wootten et al., Cell, 2016
Data in blue are from Wootten et al., Biochem Pharmacol, 2016

Supplementary Table 3. Main contacts between GLP-1R and GLP-1, exendin-4 (Ex4), oxyntomodulin (Oxyn), and exendin-P5 (ExP5), during MD simulations. Data are expressed as the occupancy (% of frames) in which the interactions were present. Black = all contacts (<25% occupancy not shown), blue = Main hydrogen bonds (side chain-side chain or side chain-backbone). Interactions are reported in order of the peptide sequence for each peptide (NB – some weaker and more transient contacts observed in the MD are not shown).

GLP-1R:GLP-1:Gs			GLP-1R:Oxyn:Gs			GLP-1R:Ex4:Gs			GLP-1R:ExP5:Gs		
**GLP-1R**	**GLP-1**	**O %**	**GLP-1R**	**Oxy**	**O %**	**GLP-1R**	**Ex4**	**O%**	**GLP-1R**	**ExP5**	**O %**
E364$^{6.53}$	H7	86.6	E364$^{6.53}$	H1	69.2	E364$^{6.53}$	H1	69.4	E364$^{6.53}$	E1	31.9
E364$^{6.53}$	H7	20.3	E364$^{6.53}$	H1	9.2	E364$^{6.53}$	H1	20.5			
E387$^{7.42}$	H7	83.4	E387$^{7.42}$	H1	72.7	E387$^{7.42}$	H1	90.9	E387$^{7.42}$	E1	38.5
						E387$^{7.42}$	H1	5.1			
R310$^{5.40}$	H7	54.1	R310$^{5.40}$	H1	48.1				R310$^{5.40}$	E1	47.3
R310$^{5.40}$	H7	21.4				R310$^{5.40}$	H1	3.4	R310$^{5.40}$	E1	45.0
Y241$^{3.44}$	H7	53.6	Y241$^{3.44}$	H1	25.6						
Y241$^{3.44}$	H7	28.2	Y241$^{3.44}$	H1	6.9						
W306$^{5.36}$	H7	29.1	W306$^{5.36}$	H1	26.1						
			W306$^{5.36}$	H1	4.8				W306$^{5.36}$	E1	7.8
I313$^{5.43}$	H7	29.1									
Q234$^{3.37}$	H7	6.5	Q234$^{3.37}$	H1	4.5						
						D372^{ECL3}	H1	31.7			
						V370$^{6.59}$	H1	26.5			
						R380$^{7.35}$	H1	3.2			
									K383$^{7.38}$	E1	20.9
E387$^{7.42}$	A8	59.4	E387$^{7.42}$	S2	89.6	E387$^{7.42}$	G2	57.4	E387$^{7.42}$	V3	38.8
			E387$^{7.42}$	S2	71.4						
L388$^{7.43}$	A8	46.0				L388$^{7.43}$	G2	30.5	L388$^{7.43}$	V3	58.9
L384$^{7.39}$	A8	39.2	L384$^{7.39}$	S2	39.0	L384$^{7.39}$	G2	57.9	L384$^{7.39}$	V3	44.8
			K383$^{7.38}$	S2	12.3						
			D372^{ECL3}	S2	11.8						
									T391$^{7.46}$	V3	37.3
R190$^{2.60}$	E9	99.9				R190$^{2.60}$	E3	79.3	R190$^{2.60}$	D4	25.5
R190$^{2.60}$	E9	99.8	R190$^{2.60}$	Q3	9.1	R190$^{2.60}$	E3	77.0	R190$^{2.60}$	D4	24.6
Y152$^{1.47}$	E9	97.1				Y152$^{1.47}$	E3	75.6	Y152$^{1.47}$	D4	37.6
Y152$^{1.47}$	E9	94.7	Y152$^{1.47}$	Q3	5.7	Y152$^{1.47}$	E3	74.7	Y152$^{1.47}$	D4	35.7
Y148$^{1.43}$	E9	56.5	Y148$^{1.43}$	Q3	41.6	Y148$^{1.43}$	E3	86.4	Y148$^{1.43}$	D4	62.0
Y148$^{1.43}$	E9	52.2	Y148$^{1.43}$	Q3	13.5	Y148$^{1.43}$	E3	84.4	Y148$^{1.43}$	D4	21.9
T391$^{7.46}$	E9	47.4	T391$^{7.46}$	Q3	37.5	T391$^{7.46}$	E3	55.5			
			T391$^{7.46}$	Q3	10.8	T391$^{7.46}$	E3	8.2			
L388$^{7.43}$	E9	34.1	L388$^{7.43}$	Q3	28.0	L388$^{7.43}$	E3	47.8	L388$^{7.43}$	D4	25.3
			E387$^{7.42}$	Q3	42.0						
			E387$^{7.42}$	Q3	18.7						
									K197$^{2.67}$	D4	26.6
									K197$^{2.67}$	D4	21.3
D372^{ECL3}	T11	11.6	D372^{ECL3}	T5	16.6						
R380$^{7.35}$	T11	9.9	R380$^{7.35}$	T5	3.3	R380$^{7.35}$	T5	12.3			
			W306$^{5.36}$	T5	5.8						
						E373^{ECL3}	T5	3.1			
						H374^{ECL3}	T5	3.1			
									Q234$^{3.37}$	N5	4.2
L388$^{7.43}$	F12	66.3	L388$^{7.43}$	F6	73.4	L388$^{7.43}$	F6	84.6			
L144$^{1.39}$	F12	57.3	L144$^{1.39}$	F6	47.8	L144$^{1.39}$	F6	60.7	L144$^{1.39}$	V7	47.2
Y148$^{1.43}$	F12	51.0				Y148$^{1.43}$	F6	56.2			
L141$^{1.36}$	F12	47.2	L141$^{1.36}$	F6	46.8	L141$^{1.36}$	F6	57.2	L141$^{1.36}$	V7	46.9

Y145$^{1.40}$	F12	47.2	Y145$^{1.40}$	F6	40.9	Y145$^{1.40}$	F6	59.7			
K197$^{2.67}$	T13	69.8				K197$^{2.67}$	T7	43.3			
K197$^{2.67}$	T13	40.7	K197$^{2.67}$	T7	4.6	K197$^{2.67}$	T7	31.2			
T298^{ECL2}	T13	29.1									
			Y145$^{1.40}$	T7	5.5						
R299^{ECL2}	S14	58.8									
						R299^{ECL2}	S8	6.3			
T298^{ECL2}	S14	56.4									
N300^{ECL2}	S14	32.2				N300^{ECL2}	S8	27.7			
						N300^{ECL2}	S8	4.8			
						E373^{ECL3}	S8	3.1			
R380$^{7.35}$	D15	38.0									
R380$^{7.35}$	D15	37.7	R380$^{7.35}$	D9	10.0	R380$^{7.35}$	D9	7.0			
						R376^{ECL3}	D9	81.5			
						R376^{ECL3}	D9	81.5			
			L141$^{1.36}$	D9	25.9	L141$^{1.36}$	D9	43.4	L141$^{1.36}$	D10	58.5
S136^{ECD}	D15	5.3				S136^{ECD}	D9	8.2	S136^{ECD}	D10	3.8
Y145$^{1.40}$	V16	26.5	Y145$^{1.40}$	Y10	89.7	Y145$^{1.40}$	L10	76.9	Y145$^{1.40}$	L11	75.5
			L142$^{1.37}$	Y10	35.9				L142$^{1.37}$	L11	27.7
			L141$^{1.36}$	Y10	29.6				L141$^{1.36}$	L11	40.2
			K202^{ECL1}	Y10	8.1						
R299^{ECL2}	S17	72.7									
R299^{ECL2}	S17	68.0	R299^{ECL2}	S11	5.7				R299^{ECL2}	S12	6.0
T298^{ECL2}	S17	68.9									
T298^{ECL2}	S17	59.8				T298^{ECL2}	S11	3.5			
L201$^{2.71}$	S17	37.7									
Y205^{ECL1}	S17	34.7							Y205^{ECL1}	S12	41.6
Y205^{ECL1}	S17	10.3	Y205^{ECL1}	S11	6.0	Y205^{ECL1}	S11	15.4	Y205^{ECL1}	S12	3.1
R299^{ECL2}	S18	46.0							R299^{ECL2}	K13	29.4
R299^{ECL2}	S18	3.7									
			V30^{ECD}	K12	29.5	V30^{ECD}	K12	43.7	V30^{ECD}	K13	59.1
									V30^{ECD}	K13	11.5
						E373^{ECL3}	K12	3.7			
L141$^{1.36}$	Y19	51.4	L141$^{1.36}$	Y13	54.6	L141$^{1.36}$	Q13	50.1			
E138$^{1.33}$	Y19	43.0	E138$^{1.33}$	Y13	61.3	E138$^{1.33}$	Q13	66.8	E138$^{1.33}$	Q14	50.4
E138$^{1.33}$	Y19	12.2	E138$^{1.33}$	Y13	11.7	E138$^{1.33}$	Q13	35.8	E138$^{1.33}$	Q14	18.7
									S136^{ECD}	Q14	68.2
									S136^{ECD}	Q14	7.8
						R134^{ECD}	Q13	6.0	S135^{ECD}	Q14	5.6
Y205^{ECL1}	L20	78.7	Y205^{ECL1}	L14	26.7	Y205^{ECL1}	M14	71.5	Y205^{ECL1}	M15	85.4
L201$^{2.71}$	L20	33.8				L201$^{2.71}$	M14	39.8			
						K202^{ECL1}	M14	47.9			
R299^{ECL2}	E21	96.1				R299^{ECL2}	E15	36.2	R299^{ECL2}	E16	59.6
R299^{ECL2}	E21	95.9	R299^{ECL2}	D15	19.5	R299^{ECL2}	E15	36.0	R299^{ECL2}	E16	59.5
Y205^{ECL1}	E21	87.4	Y205^{ECL1}	D15	40.2	Y205^{ECL1}	E15	71.4	Y205^{ECL1}	E16	81.5
Y205^{ECL1}	E21	50.0	Y205^{ECL1}	D15	35.2	Y205^{ECL1}	E15	19.2	Y205^{ECL1}	E16	30.9
L32^{ECD}	E21	82.0	L32^{ECD}	D15	59.5	L32^{ECD}	E15	73.9	L32^{ECD}	E16	97.8
S31^{ECD}	E21	79.7	S31^{ECD}	D15	59.5	S31^{ECD}	E15	91.1	S31^{ECD}	E16	88.5
S31^{ECD}	E21	50.9	S31^{ECD}	D15	36.5	S31^{ECD}	E15	28.9	S31^{ECD}	E16	21.8
			V30^{ECD}	D15	40.3	V30^{ECD}	E15	47.0	V30^{ECD}	E16	36.8

Q221^{ECL1}	E21	7.2									
W33^{ECD}	E21	5.1									
						T298^{ECL2}	E15	3.0			
V30^{ECD}	G22	43.2	V30^{ECD}	S16	71.8	V30^{ECD}	E16	32.2	V30^{ECD}	E17	44.0
			V30^{ECD}	S16	13.4						
						R134^{ECD}	E16	33.1			
						R134^{ECD}	E16	33.0	R134^{ECD}	E17	11.3
						R131^{ECD}	E16	15.4	R131^{ECD}	E17	8.3
						K130^{ECD}	E16	11.0			
						S129^{ECD}	E16	3.1	S135^{ECD}	E17	7.5
			E128^{ECD}	R17	37.7						
E138$^{1.33}$	Q23	6.4	E128^{ECD}	R17	37.6						
						K202^{ECL1}	E17	30.9			
						K202^{ECL1}	E17	29.1			
			E138$^{1.33}$	R17	8.2						
									R131^{ECD}	E18	56.3
									R131^{ECD}	E18	54.7
									R134^{ECD}	E18	7.7
Y205^{ECL1}	A24	44.8	Y205^{ECL1}	R18	53.5	Y205^{ECL1}	A18	62.0	Y205^{ECL1}	A19	62.2
L32^{ECD}	A24	32.6	L32^{ECD}	R18	38.9	L32^{ECD}	A18	37.5			
W214^{ECL1}	A24	28.6									
						A209^{ECL1}	A18	47.0			
			D198$^{2.68}$	R18	15.2						
L32^{ECD}	A25	49.8	L32^{ECD}	A19	49.8	L32^{ECD}	V19	75.3	L32^{ECD}	V20	81.5
V30^{ECD}	A25	31.4	V30^{ECD}	A19	41.5	V30^{ECD}	V19	31.5	V30^{ECD}	V20	33.4
			T35^{ECD}	A19	42.7	T35^{ECD}	V19	36.9	T35^{ECD}	V20	47.0
			S31^{ECD}	A19	26.5						
			P90^{ECD}	A19	25.3	P90^{ECD}	V19	41.9	P90^{ECD}	V20	31.3
E128^{ECD}	K26	76.5	E128^{ECD}	Q20	38.2	E128^{ECD}	R20	42.8	E128^{ECD}	R21	83.7
E128^{ECD}	K26	75.1	E128^{ECD}	Q20	9.5	E128^{ECD}	R20	39.3	E128^{ECD}	R21	83.5
W91^{ECD}	K26	27.6	W91^{ECD}	Q20	26.2	W91^{ECD}	R20	55.9			
			P90^{ECD}	Q20	31.0						
						E127^{ECD}	R20	58.8			
						E127^{ECD}	R20	57.1			
									R131^{ECD}	R21	60.3
E138$^{1.33}$	K26	12.1									
						S129^{ECD}	R20	7.7			
W214^{ECL1}	E27	30.1							W214^{ECL1}	L22	47.1
						A209^{ECL1}	L21	31.7	A209^{ECL1}	L22	52.9
Q210^{ECL1}	E27	33.2				Q210^{ECL1}	L21	29.6	Q210^{ECL1}	L22	33.6
Q210^{ECL1}	E27	12.8									
V36^{ECD}	F28	58.0	V36^{ECD}	F22	52.7	V36^{ECD}	F22	42.3	V36^{ECD}	F23	57.2
W214^{ECL1}	F28	57.5	W214^{ECL1}	F22	45.6				W214^{ECL1}	F23	61.7
T35^{ECD}	F28	28.6				T35^{ECD}	F22	37.5			
			L32^{ECD}	F22	53.4						
			W39^{ECD}	F22	25.2	W39^{ECD}	F22	59.4	W39^{ECD}	F23	33.3
L89^{ECD}	I29	67.2	L89^{ECD}	V23	48.9	L89^{ECD}	I23	46.2	L89^{ECD}	I24	75.6
P90^{ECD}	I29	46.3							P90^{ECD}	I24	25.2
W91^{ECD}	I29	45.5				W91^{ECD}	I23	28.9	W91^{ECD}	I24	30.4
Y88^{ECD}	I29	31.3									
						L123^{ECD}	I23	33.4			
									Y69^{ECD}	I24	30.3

			E127^{ECD}	Q24	4.8						
									K130^{ECD}	E25	7.1
									R131^{ECD}	E25	3.1
H212^{ECL1}	W31	70.3	H212^{ECL1}	W25	59.9	H212^{ECL1}	W25	42.6	H212^{ECL1}	W26	27.7
H212^{ECL1}	W31	4.6	H212^{ECL1}	W25	5.0						
W214^{ECL1}	W31	57.6	W214^{ECL1}	W25	26.9				W214^{ECL1}	W26	96.9
W214^{ECL1}	W31	3.3									
						Q210^{ECL1}	W25	65.8			
						Q210^{ECL1}	W25	3.0			
						Q213^{ECL1}	W25	33.8			
W39^{ECD}	L32	85.5	W39^{ECD}	L26	64.4	W39^{ECD}	L26	62.4	W39^{ECD}	L27	92.6
E68^{ECD}	L32	72.0	E68^{ECD}	L26	48.2				E68^{ECD}	L27	62.6
Y88^{ECD}	L32	29.4	Y88^{ECD}	L26	28.8	Y88^{ECD}	L26	54.6	Y88^{ECD}	L27	59.6
						R121^{ECD}	L26	38.8			
						Y69^{ECD}	L26	35.5	Y69^{ECD}	L27	34.8
R121^{ECD}	V33	97.4	R121^{ECD}	M27	70.6	R121^{ECD}	K27	77.5	R121^{ECD}	K28	95.4
Y69^{ECD}	V33	77.9	Y69^{ECD}	M27	78.3				Y69^{ECD}	K28	80.3
L123^{ECD}	V33	39.1	L123^{ECD}	M27	67.2	L123^{ECD}	K27	33.5	L123^{ECD}	K28	38.9
						E127^{ECD}	K27	69.0	E127^{ECD}	K28	87.8
						E127^{ECD}	K27	68.8	E127^{ECD}	K28	86.2
						D122^{ECD}	K27	3.4			
						K113^{ECD}	N28	3.3			
			R121 ^{ECD}	N28	9.1						
L118^{ECD}	K34	26.7									
						R121^{ECD}	G29	33.6			
									E68^{ECD}	G30	50.5
			E68^{ECD}	T29	7.9						
			E68^{ECD}	K30	37.3				E68^{ECD}	G31	43.2
E68^{ECD}	R36	96.3	E68^{ECD}	K30	36.4						
H212^{ECL1}	R36	16.5									
									D67^{ECD}	G31	25.4
			E68^{ECD}	R31	51.0						
			E68^{ECD}	R31	42.4						
			E127^{ECD}	R31	22.2						
			S124^{ECD}	R31	7.6						
H212^{ECL1}	NH2	5.8	H212^{ECL1}	R31	7.1						
			D122^{ECD}	R31	4.9						
			Q112^{ECD}	N32	6.3						
						R121^{ECD}	S32	6.5			
						D67^{ECD}	S32	4.6			
									E68^{ECD}	S33	7.8

Supplementary Table 4. Main contacts between GLP-1R (in complex with GLP-1, Ex4, Oxyn, and ExP5) and the G protein Gα subunit, during MD simulations. Data are expressed as the occupancy (% of frames) in which the interactions were present. Black = all contacts (<15% occupancy not shown), blue = main hydrogen bonds (side chain-side chain or side chain-backbone - (NB – some weaker and more transient contacts observed in the MD are not shown). Interactions are listed by regions within the G protein (using the CGN nomenclature).

GLP-1R:GLP-1:Gs			GLP-1R:Oxyn:Gs			GLP-1R:Ex4:Gs			GLP-1R:ExP5:Gs		
**GLP-1R**	**Gα**	**O %**	**GLP-1R**	**Gα**	**O %**	**GLP-1R**	**Gα**	**O%**	**GLP-1R**	**Gα**	**O %**
					**INTERACTIONS of αN**						
			E262$^{4.38}$	K32	3.3						
						E262$^{4.38}$	K34	15.0			
E262$^{4.38}$	K34	10.7	E262$^{4.38}$	K34	12.6	E262$^{4.38}$	K34	13.8	E262$^{4.38}$	K34	11
						Q263$^{4.39}$	K34	2.0			
F260^{ICL2}	Q35	23.1	F260^{ICL2}	Q35	33.6	F260^{ICL2}	Q35	51.7			
S261^{ICL2}	Q35	34.2	S261^{ICL2}	Q35	18.4	S261^{ICL2}	Q35	32.8			
S261^{ICL2}	Q35	11.3				S261^{ICL2}	Q35	14.9	S261^{ICL2}	Q35	2.4
			E262$^{4.38}$	Q35	6.6	E262$^{4.38}$	Q35	4.4			
Q263$^{4.39}$	Q35	2.2	Q263$^{4.39}$	Q35	8.3	Q263$^{4.39}$	Q35	2.4			
					**INTERACTIONS OF hns1/S1**						
V259^{ICL2}	R38	35.0	V259^{ICL2}	R38	25.8	V259^{ICL2}	R38	30.8	V259^{ICL2}	R38	21.8
F260^{ICL2}	R38	19.5	F260^{ICL2}	R38	20.8	F260^{ICL2}	R38	40.8			
E262$^{4.38}$	R38	83.0	E262$^{4.38}$	R38	56.3	E262$^{4.38}$	R38	77.9	E262$^{4.38}$	R38	56.6
E262$^{4.38}$	R38	82.1	E262$^{4.38}$	R38	55.5	E262$^{4.38}$	R38	77.0	E262$^{4.38}$	R38	55.5
F260^{ICL2}	A39	26.5	F260^{ICL2}	A39	21.4	F260^{ICL2}	A39	57.8			
			V259^{ICL2}	A39	16.9				V259^{ICL2}	A39	21.8
			E423$^{7.78}$	R42	39.7						
					**INTERACTIONS OF α5**						
									V331$^{5.61}$	L394	27.9
K334$^{5.64}$	L394	33.9	K334$^{5.64}$	L394	71.6	K334$^{5.64}$	L394	41.1	K334$^{5.64}$	L394	32.0
L335$^{5.65}$	L394	46.9	L335$^{5.65}$	L394	71.1	L335$^{5.65}$	L394	39.5	L335$^{5.65}$	L394	46.5
			N338$^{5.68}$	L394	52.2						
R348$^{6.37}$	L394	100	R348$^{6.37}$	L394	96.2	R348$^{6.37}$	L394	88.8	R348$^{6.37}$	L394	80.8
R348$^{6.37}$	L394	100.0	R348$^{6.37}$	L394	96.1	R348$^{6.37}$	L394	88.3	R348$^{6.37}$	L394	66.7
K342$^{6.31}$	L394	2.3				K342$^{6.31}$	L394	5.0			
									K351$^{6.40}$	L394	27.3
									K351$^{6.40}$	L394	8.9
V327$^{5.57}$	L393	17.1							V327$^{5.57}$	L393	17.9
V331$^{5.61}$	L393	74.3	V331$^{5.61}$	L393	72.6	V331$^{5.61}$	L393	72.8	V331$^{5.61}$	L393	31.8
S352$^{6.41}$	L393	73.4	S352$^{6.41}$	L393	93.2	S352$^{6.41}$	L393	82.8	S352$^{6.41}$	L393	91.5
									L251$^{3.54}$	L393	20.9
			T355$^{6.44}$	L393	31.9	T355$^{6.44}$	L393	22.8	T355$^{6.44}$	L393	31.8
L356$^{6.45}$	L393	36.3	L356$^{6.45}$	L393	41.8	L356$^{6.45}$	L393	41.5	L356$^{6.45}$	L393	39.8
R348$^{6.37}$	E392	94.3	R348$^{6.37}$	E392	65.3	R348$^{6.37}$	E392	68.0			
R348$^{6.37}$	E392	91.8	R348$^{6.37}$	E392	53.1	R348$^{6.37}$	E392	63.4	R348$^{6.37}$	E392	3.4
			K351$^{6.40}$	E392	33.9	K351$^{6.40}$	E392	22.1	K351$^{6.40}$	E392	49.0
K351$^{6.40}$	E392	2.1	K351$^{6.40}$	E392	8.5	K351$^{6.40}$	E392	18.2	K351$^{6.40}$	E392	40.4
S352$^{6.41}$	E392	22.7				S352$^{6.41}$	E392	21.4			
									V405$^{7.60}$	E392	16.0
						N406$^{7.61}$	E392	2.8			

N407$^{7.62}$	E392	20.0				N407$^{7.62}$	E392	16.6	N407$^{7.62}$	E392	25.5
N407$^{7.62}$	E392	12.0	N407$^{7.62}$	E392	7.8	N407$^{7.62}$	E392	8.7			
R176$^{2.46}$	Y391	32.9	R176$^{2.46}$	Y391	39.8	R176$^{2.46}$	Y391	41.1	R176$^{2.46}$	Y391	33.3
						R176$^{2.46}$	Y391	7.0			
Y250$^{3.53}$	Y391	34.9	Y250$^{3.53}$	Y391	69.2	Y250$^{3.53}$	Y391	28.1	Y250$^{3.53}$	Y391	45.4
						Y250$^{3.53}$	Y391	4.1	Y250$^{3.53}$	Y391	2.9
L251$^{3.54}$	Y391	53.2	L251$^{3.54}$	Y391	51.4	L251$^{3.54}$	Y391	46.4	L251$^{3.54}$	Y391	57.0
L254$^{6.43}$	Y391	41.4	L254$^{6.43}$	Y391	46.5	L254$^{6.43}$	Y391	34.0	L254$^{6.43}$	Y391	53.8
Y402$^{7.57}$	Y391	35.2	Y402$^{7.57}$	Y391	48.8				Y402$^{7.57}$	Y391	31.4
						R176$^{2.46}$	Q390	20.0	R176$^{2.46}$	Q390	18.6
R176$^{2.46}$	Q390	2.0				R176$^{2.46}$	Q390	2.1	R176$^{2.46}$	Q390	2.6
N407$^{7.62}$	Q390	17.3									
N407$^{7.62}$	Q390	9.9				N407$^{7.62}$	Q390	2.3	N407$^{7.62}$	Q390	7.0
E408$^{7.63}$	Q390	7.6									
						D344$^{6.33}$	R389	16.9			
						D344$^{6.33}$	R389	16.9			
R348$^{6.37}$	R389	65.9	R348$^{6.37}$	R389	49.5	R348$^{6.37}$	R389	25.1	R348$^{6.3}$	R389	18.0
						R348$^{6.37}$	R389	2.6			
L255$^{3.58}$	L388	41.2	L255$^{3.58}$	L388	39.6	L255$^{3.58}$	L388	48.0	L255$^{3.58}$	L388	42.7
V331$^{5.61}$	L388	57.5	V331$^{5.61}$	L388	49.9	V331$^{5.61}$	L388	49.6	V331$^{5.61}$	L388	39.8
						I330$^{5.60}$	L388	15.4			
K334$^{5.64}$	L388	25.6	K334$^{5.64}$	L388	37.8	K334$^{5.64}$	L388	27.6			
L254$^{6.43}$	H387	85.5	L254$^{6.43}$	H387	91.5	L254$^{6.43}$	H387	86.1	L254$^{6.43}$	H387	82.1
L255$^{3.58}$	H387	29.1	L255$^{3.58}$	H387	29.4	L255$^{3.58}$	H387	26.1	L255$^{3.58}$	H387	34.1
									S258^{ICL2}	H387	3.0
K334$^{5.64}$	R385	57.9	K334$^{5.64}$	R385	42.8	K334$^{5.64}$	R385	57.7	K334$^{5.64}$	R385	66.2
A337$^{5.67}$	R385	44.3	A337$^{5.67}$	R385	80.4	A337$^{5.67}$	R385	51.8	A337$^{5.67}$	R385	15.4
N338$^{5.68}$	R385	55.8	N338$^{5.68}$	R385	95.8	N338$^{5.68}$	R385	41.7	N338$^{5.68}$	R385	64.3
N338$^{5.68}$	R385	51.5	N338$^{5.68}$	R385	94.9	N338$^{5.68}$	R385	22.9	N338$^{5.68}$	R385	55.6
L255$^{3.58}$	Q384	94.5	L255$^{3.58}$	Q384	84.1	L255$^{3.58}$	Q384	94.8	L255$^{3.58}$	Q384	88.2
I330$^{5.60}$	Q384	18.0				I330$^{5.60}$	Q384	19.0	I330$^{5.60}$	Q384	23.0
K334$^{5.64}$	Q384	79.1	K334$^{5.64}$	Q384	48.3	K334$^{5.64}$	Q384	80.6	K334$^{5.64}$	Q384	53.1
K334$^{5.64}$	Q384	55.1	K334$^{5.64}$	Q384	20.1	K334$^{5.64}$	Q384	52.0	K334$^{5.64}$	Q384	31.4
K334$^{5.64}$	D381	100	K334$^{5.64}$	D381	100	K334$^{5.64}$	D381	100	K334$^{5.64}$	D381	98.6
K334$^{5.64}$	D381	99.8	K334$^{5.64}$	D381	99.8	K334$^{5.64}$	D381	99.8	K334$^{5.64}$	D381	97.4
						N338$^{5.68}$	D381	4.3	K336$^{5.66}$	D381	4.7
L254$^{6.43}$	R380	41.0				L254$^{6.43}$	R380	42.0			
L255$^{3.58}$	R380	64.9	L255$^{3.58}$	R380	49.2	L255$^{3.58}$	R380	73.0	L255$^{3.58}$	R380	39.6
F257$^{3.60}$	R380	50.4	F257$^{3.60}$	R380	17.3	F257$^{3.60}$	R380	49.0	F257$^{3.60}$	R380	18.1
S258^{ICL2}	R380	39.6	S258^{ICL2}	R380	36.3	S258^{ICL2}	R380	41.7	S258^{ICL2}	R380	41.7
S258^{ICL2}	R380	5.3	S258^{ICL2}	R380	14.2	S258^{ICL2}	R380	7.5	S258^{ICL2}	R380	8.8
A256$^{3.59}$	R380	25.1	A256$^{3.59}$	R380	33.5	A256$^{3.59}$	R380	26.7	A256$^{3.59}$	R380	20.6
									K336$^{5.66}$	D378	3.2

INTERACTIONS OF s2s3/S3

			F260^{ICL2}	K216	29.1						
S258^{ICL2}	V217	36.1	S258^{ICL2}	V217	30.4	S258^{ICL2}	V217	35.1	S258^{ICL2}	V217	17.0
									V259^{ICL2}	V217	15.0

**INTERACTIONS OF H3**											
			C341$^{6.30}$	W277	15.4						
**INTERACTIONS OF H4**											
									K342$^{6.31}$	E322	17.2
K342$^{6.31}$	E322	5.3							K342$^{6.31}$	E322	16.3
									K342$^{6.31}$	D323	20.3
									K342$^{6.31}$	D323	19.7
						L339$^{5.69}$	R342	15.5	L339$^{5.69}$	R342	19.2
									M340^{ICL3}	R342	18.9
									K342$^{6.31}$	D343	23.6
									K342$^{6.31}$	D343	23.1
L339$^{5.69}$	L346	18.2	L339$^{5.69}$	L346	33.0	L339$^{5.69}$	L346	35.8			
M340^{ICL3}	L346	17.7							M340^{ICL3}	L346	36.7
									C341$^{6.30}$	L346	36.1
**INTERACTIONS OF h4s6**											
									C341$^{6.30}$	I348	15.4
			L339$^{5.69}$	S349	36.0						
						C341$^{6.30}$	S349	27.0			
			L339$^{5.69}$	T350	38.1						
			C341$^{6.30}$	T350	65.8	C341$^{6.30}$	T350	37.0			
						T343$^{6.32}$	T350	2.4			
			C341$^{6.30}$	S352	34.8						
						K342$^{6.31}$	S352	17.1			
			C341$^{6.30}$	G353	35.8						
						K342$^{6.31}$	G353	23.2			
			C341$^{6.30}$	D354	36.6						
									R348$^{6.37}$	D354	24.3
			R348$^{6.37}$	D354	14.6	R348$^{6.37}$	D354	8.3	R348$^{6.37}$	D354	23.7
						K342$^{6.31}$	D354	3.3			
									A337$^{5.67}$	Y358	17.6
N338$^{5.68}$	Y358	18.0	N338$^{5.68}$	Y358	73.4				N338$^{5.68}$	Y358	32.8
N338$^{5.68}$	Y358	2.8	N338$^{5.68}$	Y358	2.3				N338$^{5.68}$	Y358	6.4
									C341$^{6.30}$	Y358	22.3
**INTERACTIONS OF S6**											
									A337$^{5.67}$	Y360	22.2
						N338$^{5.68}$	Y360	15.0	N338$^{5.68}$	Y360	19.5
									N338$^{5.68}$	Y360	6.3
						L339$^{5.69}$	Y360	15.5			
									M340^{ICL3}	Y360	18.8
						L339$^{5.69}$	P361	20.6			
									M340^{ICL3}	P361	25.2

Supplementary Table 5. Main contacts between GLP-1R (in complex with GLP-1, Ex4, Oxyn, and ExP5) and the G protein Gβ subunit, during MD simulations. Data are expressed as the occupancy (% of frames) in which the interactions were present. Black = all contacts (<15% occupancy not shown), blue = main hydrogen bonds (side chain-side chain or side chain-backbone) - (NB – some weaker and more transient contacts observed in the MD are not shown). Interactions are listed in order of the sequence of the Gβ subunit.

GLP-1R:GLP-1:Gs			GLP-1R:Oxyn:Gs			GLP-1R: Ex4:Gs			GLP-1R:ExP5:Gs		
GLP-1R	Gβ	O %	GLP-1R	Gβ	O %	GLP-1R	Gβ	O %	GLP-1R	Gβ	O %
E423$^{7.78}$	R42	52.5	E423$^{7.78}$	R42	39.7	E423$^{7.78}$	R42	25.4	E423$^{7.78}$	R42	55.1
E423$^{7.78}$	R42	40.1	E423$^{7.78}$	R42	24.7	E423$^{7.78}$	R42	16.8	E423$^{7.78}$	R42	44.8
E423$^{7.78}$	Q44	18.4									
E423$^{7.78}$	Q44	7.5	E423$^{7.78}$	Q44	2.7	E423$^{7.78}$	Q44	2.7			
			L422$^{7.77}$	R46	15.2						
E423$^{7.78}$	R46	48.4	E423$^{7.78}$	R46	49.7	E423$^{7.78}$	R46	74.2	E423$^{7.78}$	R46	33.1
E423$^{7.78}$	R46	47.3	E423$^{7.78}$	R46	41.0	E423$^{7.78}$	R46	61.4	E423$^{7.78}$	R46	29.0
			H171^{ICL1}	R52	35.9	H171^{ICL1}	R52	30.0			
			H171^{ICL1}	R52	4.7	H171^{ICL1}	R52	10.2			
			E412$^{7.67}$	R52	2.1	E412$^{7.67}$	R52	8.5			
R421$^{7.76}$	D291	17.7									
R421$^{7.76}$	D291	17.7									
R414$^{7.69}$	D291	5.4									
			E418$^{7.73}$	N293	9.6				E418$^{7.73}$	N293	7.8
L422$^{7.77}$	N293	30.2				L422$^{7.77}$	N293	19.9	L422$^{7.77}$	N293	16.6
E423$^{7.78}$	R304	19.0	E423$^{7.78}$	R304	18.9						
E423$^{7.78}$	R304	14.3	E423$^{7.78}$	R304	18.5	E423$^{7.78}$	R304	4.6	E423$^{7.78}$	R304	10.8
									E423$^{7.78}$	G306	22.4
L422$^{7.77}$	V307	40.8	L422$^{7.77}$	V307	32.0	L422$^{7.77}$	V307	39.2	L422$^{7.77}$	V307	35.9
E423$^{7.78}$	V307	26.4	E423$^{7.78}$	V307	20.8				E423$^{7.78}$	V307	31.6
			R419$^{7.74}$	A309	16.4	R419$^{7.74}$	A309	30.3	R419$^{7.74}$	A309	16.2
L422$^{7.77}$	A309	37.7	L422$^{7.77}$	A309	23.7	L422$^{7.77}$	A309	41.4	L422$^{7.77}$	A309	32.0
			K415$^{7.70}$	G310	29.5						
R419$^{7.74}$	G310	49.4	R419$^{7.74}$	G310	38.0	R419$^{7.74}$	G310	49.6	R419$^{7.74}$	G310	42.8
R419$^{7.74}$	H311	63.5	R419$^{7.74}$	H311	33.3	R419$^{7.74}$	H311	39.9	R419$^{7.74}$	H311	45.4
H171^{ICL1}	D312	59.9	H171^{ICL1}	D312	16.5				H171^{ICL1}	D312	27.2
H171^{ICL1}	D312	34.6	H171^{ICL1}	D312	4.2				H171^{ICL1}	D312	22.5
K415$^{7.70}$	D312	95.1	K415$^{7.70}$	D312	99.1	K415$^{7.70}$	D312	94.8	K415$^{7.70}$	D312	93.9
K415$^{7.70}$	D312	92.6	K415$^{7.70}$	D312	98.9	K415$^{7.70}$	D312	89.7	K415$^{7.70}$	D312	91.3
R419$^{7.74}$	D312	66.8	R419$^{7.74}$	D312	39.4	R419$^{7.74}$	D312	57.9	R419$^{7.74}$	D312	50.5
R419$^{7.74}$	D312	66.4	R419$^{7.74}$	D312	39.3	R419$^{7.74}$	D312	57.5	R419$^{7.74}$	D312	50.2

Supplementary Table 6. Summary of the network analysis communities, computed on the GLP-1R:Gs complex with GLP-1, exendin-4, oxyntomodulin, and exendin-P5. The GLP-1R structural elements are colored from red (TM1 and ICL1) to black (H8). Intracellular and extracellular portion of the TM are indicated as IC and EC, respectively.

Complex	Total Number Communities	TMD communities	Number Gs Communities
**GLP-1R:GLP-1:Gs**	16	TM6(EC)/TM7/TM1/TM2/ECL3<br>TM3(IC)/TM4(IC)<br>TM5(IC)/TM6(IC)/ICL3<br>TM3(EC)/TM4(EC)/TM5(EC)/ECL1/ECL2<br>H8	9
**GLP-1R:exendin-4:Gs**	18	TM7/TM1/TM2(IC)/ICL1/H8<br>TM2(EC)/ECL1<br>TM3/TM4/TM5(EC)/ICL2<br>TM5(IC)/TM6/ICL3	12
**GLP-1R:oxyntomodulin:Gs**	14	TM7(IC)/TM1(IC)/TM2/H8<br>TM1(EC)/TM7(EC)/ECL3<br>TM3(IC)/TM4/TM5/TM6(IC)/ICL2/ICL3<br>TM3(EC)/ECL2	8
**GLP-1R:exendin-P5:Gs**	16	TM7/TM1/ICL1/TM2(IC)<br>TM2(EC)/TM3(EC)/TM4/ECL2<br>TM5/TM6/ICL3<br>TM3(IC)<br>H8	8

Supplementary Table 7: CryoEM Data collection and modelling refinement statistics.

**Data Collection**	GLP-1R:DNGs:EX4	GLP-1R:DNGs:Oxyntomodullin
Micrographs	8816	3181
Electron dose (e^-/A^2)	48	50
Voltage (kV)	300	300
Pixel size (Å)	0.87	1.06
Defocus range (µm)	0.5-1.5	0.5
Symmetry imposed	C1	C1
Particles (final map)	277,494	366,351
Resolution (0.143 FSC)(Å)	3.7	3.3
**Refinement**		
CC_{map_model}		
Map sharpening B factor (Å^2)	-137	-50
**Model Quality**		
R.m.s. deviations		
Bond length (Å)	0.006	0.002
Bond angles (°)	0.890	0.447
Ramachandran		
Favoured (%)	92.98	97.95
Outliers (%)	0	0
Rotamer outliers	0.1	0.68
C-Beta deviations (%)	0	0
Clashscore	8.28	3.05
MolProbity score	1.90	1.11
