# Supplementary material for: Dynamics of GLP-1R peptide agonist engagement are correlated with kinetics of G protein activation
Source: Nat Commun. 2022 Jan 10;13:92. doi: 10.1038/s41467-021-27760-0 (PMC8748714; doi:10.1038/s41467-021-27760-0)
Supplement: Supplementary file 3 — Description of Additional Supplementary Files [file 41467_2021_27760_MOESM3_ESM.pdf]

Description of Additional Supplementary Files

File name: Supplementary Movie 1

Description: MD simulations of the peptide bound GLP1R:Gs complexes. Comparison of the dynamic (merged MD replicas) of the four GLP-1R:agonist:Gs complexes. GLP-1R is shown as a purple ribbon. The residues forming intermolecular contacts and hydrogen bonds (transient red dotted lines) are shown as sticks. GLP-1, exendin-4 (Ex4), oxyntomodulin (Oxyn), and exendin-P5 (ExP5) are shown in red, green, yellow, and cyan respectively.
