# Supplementary material for: Dynamics of GLP-1R peptide agonist engagement are correlated with kinetics of G protein activation
Source: Nat Commun. 2022 Jan 10;13:92. doi: 10.1038/s41467-021-27760-0 (PMC8748714; doi:10.1038/s41467-021-27760-0)
Supplement: Supplementary file 5 — Reporting Summary [file 41467_2021_27760_MOESM5_ESM.pdf]

nature portfolio

Corresponding author(s): Denise Wootten

Last updated by author(s): Nov 2, 2021

Reporting Summary

Nature Portfolio wishes to improve the reproducibility of the work that we publish. This form provides structure for consistency and transparency in reporting. For further information on Nature Portfolio policies, see our Editorial Policies and the Editorial Policy Checklist.

Statistics

For all statistical analyses, confirm that the following items are present in the figure legend, table legend, main text, or Methods section.

n/a	Confirmed	
☐	☒	The exact sample size (n) for each experimental group/condition, given as a discrete number and unit of measurement
☒	☐	A statement on whether measurements were taken from distinct samples or whether the same sample was measured repeatedly
☐	☒	The statistical test(s) used AND whether they are one- or two-sided<br>*Only common tests should be described solely by name; describe more complex techniques in the Methods section.*
☐	☒	A description of all covariates tested
☐	☒	A description of any assumptions or corrections, such as tests of normality and adjustment for multiple comparisons
☐	☒	A full description of the statistical parameters including central tendency (e.g. means) or other basic estimates (e.g. regression coefficient) AND variation (e.g. standard deviation) or associated estimates of uncertainty (e.g. confidence intervals)
☐	☒	For null hypothesis testing, the test statistic (e.g. F, t, r) with confidence intervals, effect sizes, degrees of freedom and P value noted<br>*Give P values as exact values whenever suitable.*
☒	☐	For Bayesian analysis, information on the choice of priors and Markov chain Monte Carlo settings
☒	☐	For hierarchical and complex designs, identification of the appropriate level for tests and full reporting of outcomes
☐	☒	Estimates of effect sizes (e.g. Cohen's d, Pearson's r), indicating how they were calculated

Our web collection on statistics for biologists contains articles on many of the points above.

Software and code

Policy information about availability of computer code

Data collection	SerialEM, BMG Pherastar reader controls and MARS analysis software
Data analysis	GraphPad Prism 9.0, UCSF Chimera v1.14, ChimeraX v1.0, VMD, GetCOntacts analysis tool (https://getcontacts.github.io/), avconv (https://libav.org/avconv.html), AquaMMapS, ACEMD, Modeller, MDFF, Coot, Phenix, Chem3D, Relion 3.0, Relion 2.03, MotionCor2, Gctf, EMAN2, Network and Community analysis (http://faculty.scs.illinois.edu/schulten/software/networkTools/index.html), ShinyCircos (https://venyao.xyz/shinyCircos/), gautomatch (http://www.mrc-lmb.cam.ac.uk/kzhang/Gautomatch/).

For manuscripts utilizing custom algorithms or software that are central to the research but not yet described in published literature, software must be made available to editors and reviewers. We strongly encourage code deposition in a community repository (e.g. GitHub). See the Nature Portfolio guidelines for submitting code & software for further information.

Data

Policy information about availability of data

All manuscripts must include a data availability statement. This statement should provide the following information, where applicable:

- Accession codes, unique identifiers, or web links for publicly available datasets
- A description of any restrictions on data availability
- For clinical datasets or third party data, please ensure that the statement adheres to our policy

All relevant data are included with the manuscript, source data or Supplemental Information. Atomic coordinates and the cryo-EM density map have been deposited in the Protein Data Bank (PDB) under accession numbers 7LLY and 7LLL, and EMDB entry IDs EMD-23436 and EMD-23425, for oxyntomodulin and exendin-4, respectively. The MD trajectories have been deposited on Zenodo (https://zenodo.org/record/52262 09). Source data are provided with this paper.

March 2021

Atomic coordinates for structures not determined in this study, but used as the starting structure for the MD are available from the PDB under accession codes 6B3J and 6X18.

Field-specific reporting

Please select the one below that is the best fit for your research. If you are not sure, read the appropriate sections before making your selection.

☒ Life sciences ☐ Behavioural & social sciences ☐ Ecological, evolutionary & environmental sciences

For a reference copy of the document with all sections, see nature.com/documents/nr-reporting-summary-flat.pdf

Life sciences study design

All studies must disclose on these points even when the disclosure is negative.

Sample size	For each GPCR complex, one cryo-EM dataset comprising several thousand micrographs was collected. The number of micrographs in each dataset was determined based on the available microscope time and our experience with GPCR complexes. No statistical methods were used to predetermine sample size. All functional data were obtained from at least five independent experiments to ensure each data point was repeatable.
Data exclusions	In accordance with standard cryo-EM practice, micrographs with low estimated CTF resolution were excluded from further processing. No data were excluded from the pharmacological assays.
Replication	Each cryo-EM dataset comprises millions of copies of the investigated GPCR complex and therefore has inherent replication by using random particle subsets during the analyses. For pharamcological assays, each individual experiment was performed in duplicate or triplicate. All findings were reliably reproduced
Randomization	Cryo-EM - Random particle subsets were used during the 3D auto-refinement steps in Relion. While randomisation was not performred for pharmacology experiments, plates layouts were altered for each independent experiment such that the location of different cell lines or drug dilutions differed in the different experimental repeats. No animals or human research participants are involved in this study.
Blinding	Blinding was not performed in this study, as protein samples are not required to be allocated into experimental groups in protein structural studies, and no animals or human research participants are involved in this study.

Reporting for specific materials, systems and methods

We require information from authors about some types of materials, experimental systems and methods used in many studies. Here, indicate whether each material, system or method listed is relevant to your study. If you are not sure if a list item applies to your research, read the appropriate section before selecting a response.

Materials & experimental systems

n/a	Involved in the study	
☐	☒	Antibodies
☐	☒	Eukaryotic cell lines
☒	☐	Palaeontology and archaeology
☒	☐	Animals and other organisms
☒	☐	Human research participants
☒	☐	Clinical data
☒	☐	Dual use research of concern

Methods

n/a	Involved in the study	
☒	☐	ChIP-seq
☒	☐	Flow cytometry
☒	☐	MRI-based neuroimaging

Antibodies

Antibodies used	Ml anti-FLAG (generated in house)<br>cMyc mouse clone 9E10 (generated in house)<br>Rabbit anti-Gs C-18 antibody (cat no sc-383), Santa Cruz<br>Mouse Penta-His antibody (cat no 34660), QIAGEN<br>680RD goat anti-mouse antibody (LI-COR cat no 926-68070)<br>800CW goat anti-rabbit antibody (LI-COR cat no 926-32211)<br>Alexa Fluor 647-anti cAMP antibody (PerkinElmer - a component of the Lance assay kit - Cat No ADO264)
Validation	All antibodies were used for Western blot analysis, protein purification or ELISA were validated by the manufacturers.

March 2021

Eukaryotic cell lines

Policy information about cell lines

Cell line source(s)	Cells used in assays were obtained from ATCC (HEK293), Invitrogen (Cho-FlpIn) or expression systems (Tni)
Authentication	Grown from original stocks purchased from suppliers. Cell lines were not authenticated in house.
Mycoplasma contamination	Cells were tested regularly in our laboratory and were free of mycoplasma contamination
Commonly misidentified lines (See ICLAC register)	not relevant - No commonly misidentified cell lines were used.
